# Artificial intelligence, intellectual property, and human rights: mapping the legal landscape in European health systems

**DOI:** 10.1038/s44401-025-00050-3

**Published:** 2025-11-25

**Authors:** Robin van Kessel, Jelena Schmidt, Hannah van Kolfschooten, Sam Feudo, Katie Young, Laura Valtere, Timo Minssen, Elias Mossialos

**Affiliations:** 1 LSE Health, Department of Health Policy, London School of Economics and Political Science, London, United Kingdom; 2 Centre for Health and Healthcare, World Economic Forum, Geneva, Switzerland; 3 Department of International Health, Care and Public Health Research Institute (CAPHRI), Maastricht University, Maastricht, Netherlands; 4 Law Centre for Health and Life, University of Amsterdam, Amsterdam, Netherlands; 5 Center for Advanced Studies in Bioscience Innovation Law (CeBIL), Faculty of Law, University of Copenhagen, Copenhagen, Denmark

**Keywords:** Health policy, Medical ethics, Intellectual-property rights, Computer science

## Abstract

Intellectual property (IP) rights and IP-related rights, such as trade secrets and regulatory exclusivities, play a crucial role in the development and deployment of artificial intelligence (AI) technologies. However, possible interactions may be anticipated when comparing the legal relationships formed by these rights with those established by human rights. This study synthesises 53 laws and treaties illustrating the IP landscape for AI in health systems across Europe and examines their intersections with health-focused human rights. Our analysis reveals that a great variety of datasets, software, hardware, output, AI model architecture, data bases, and graphical user interfaces can be subject to IP protection. Although codified limitations and exceptions on IP and IP-related rights exist, interpretation of their conditions and scope permits for diverse interpretations and is left to the discretion of courts. Comparing these rights to health-focused human rights highlights tensions between promoting innovation and ensuring accessibility, quality, and equity in health systems, as well as between human rights ideals and the protection of European digital sovereignty. As these rights often pursue conflicting objectives and may involve trade-offs, future research should explore new ways to reconcile these objectives and foster solidarity in sharing the risks and benefits among stakeholders.

## Introduction

Artificial intelligence (AI) technologies refer to mathematical models that generate predictions based on the data they were trained on^1^. These technologies can be deterministic (i.e., following a set of instructions given a set of inputs to produce clearly defined outputs) or probabilistic (i.e., comparing vast pools of data and drawing inferences from the connections between data points)^2^. The term ‘AI technologies’ often describes probabilistic, large, resource-intensive machine learning systems, with so-called ‘generative’ AI technologies drawing the most attention^2^. In this article, we exclusively refer to probabilistic systems when referring to AI technologies. In the context of health systems, AI technologies can help predict incidence rates, provide disease diagnoses, and recommendations on what medical treatment to prescribe^1,3,4^. AI technologies could also have cost-saving potential for health systems by streamlining and optimising healthcare processes and providing novel modes of healthcare delivery^5–7^. However, research continues to show problems in the implementation of AI technologies within health systems^8–10^.

AI developers often rely on intellectual property (IP) rights and IP-related rights to protect investments in new healthcare technologies^11–13^. These rights enable the recovery of investment in innovation by granting developers exclusive control over their inventions for a limited time period. In case of successful commercialisation, IP rights and IP-related rights, such as patents, trade secret protection, or copyrights, allow inventors to benefit financially, motivating further investment, directing research activities, and facilitating innovation and technology transfer^14^. In the context of AI technologies, trade secrets may be used to conceal information about the AI development pipeline and the structure and contents of the training datasets used to develop AI technologies. In contrast, other exclusive rights, such as patents, require the disclosure information about the invention^8,9^. However, since such disclosure are often limited and the technology can be protected by various layers of different IP rights and IP-related rights, this can be particularly problematic in the health sector, given the importance of transparency and accountability mechanisms^8,9,15^.

Concurrently, the intersection between AI technologies and human rights has received ample attention. For instance, AI technologies and algorithmic biases can perpetuate existing biases found in training data and lead to inequitable health outcomes or discriminatory practices^16,17^. Biases in training datasets may also result in AI technologies being disproportionately developed for certain population groups, further increasing inequalities^18,19^. Concerns about autonomy and accountability may arise when AI technologies are used to make independent health-related decisions^20–22^.

Although AI-related research on IP and human rights is steadily advancing, the interactions of these rights in health systems remain unclear. As AI stakeholders become integral to the health sector^23,24^, it is important to explore how these rights can co-exist and become mutually reinforcing. In this article, we first examine how IP legislation applies to training datasets and AI technologies for health systems at the international, European Union (EU), and national levels. We acknowledge that IP laws in the EU have been highly harmonised through the TRIPS Agreement, though national legislation needs to be assessed, as health system governance remains a national competence in the EU, and the degree to which that national competence is leveraged for the IP governance of AI technologies in health systems is unclear. Second, we compare the legal relationships established by IP rights and IP-related rights to those established by health-focused human rights (i.e., the fundamental rights grounded in international human rights frameworks that aim to protect, promote, and fulfil the highest attainable standards of physical and mental health)^25^ in order to identify where conflicts between these and IP rights can arise. The health-focused human rights used in the analysis of this study are derived from the International Covenant on Economic Social and Cultural Rights (ICESCR), the International Covenant on Civil and Political Rights (ICCPR), the European Convention on Human Rights (ECHR), and the Framework Convention on AI and Human Rights, Democracy, and the Rule of Law (FCAI).

## Results

**Fig. 1:**
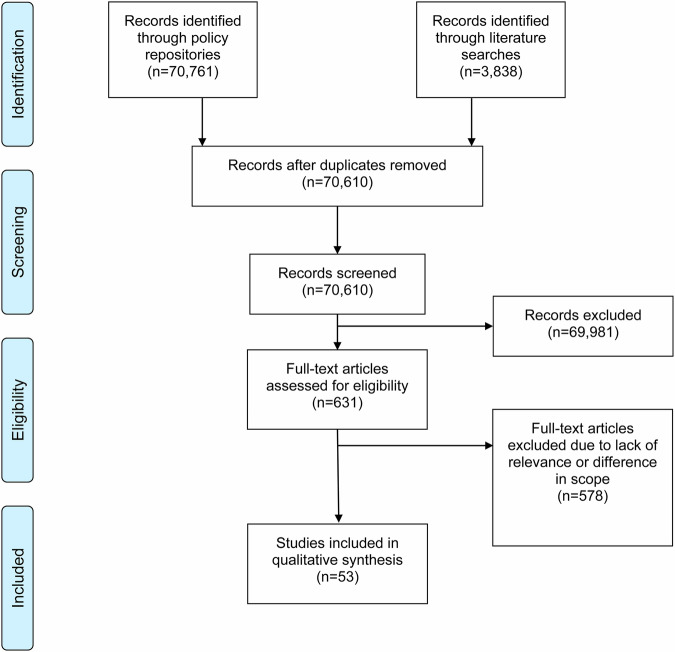
PRISMA chart illustrating the data collection process.

**Fig. 2:**
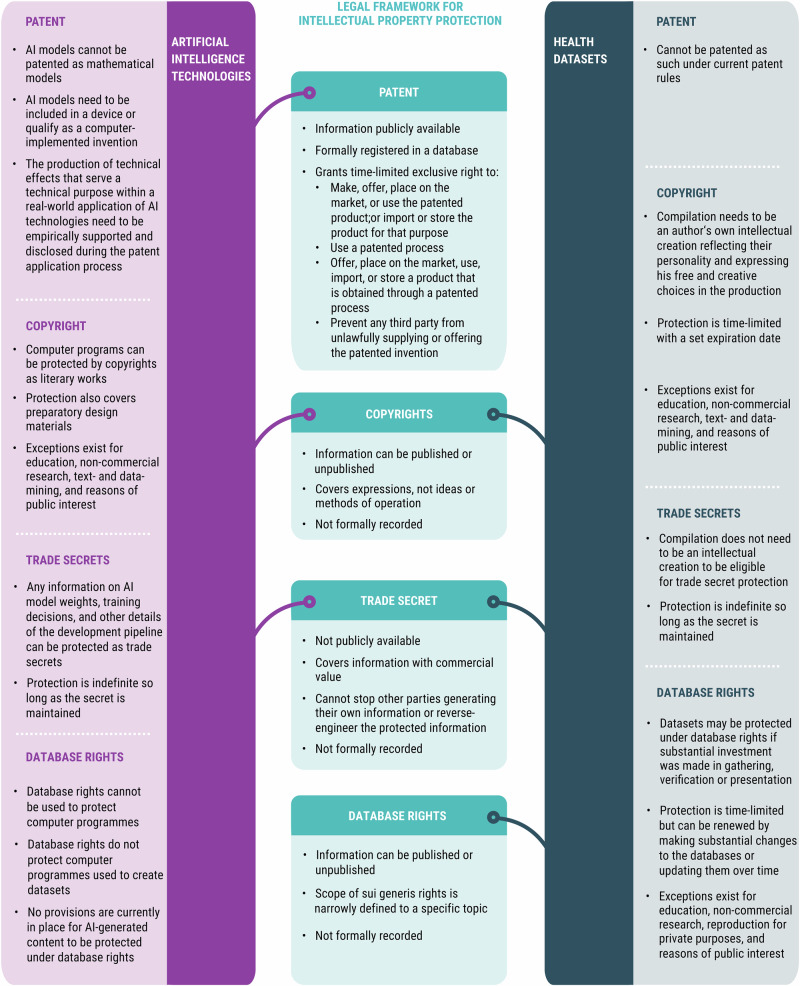
Legal framework for selected IP rights and IP-related rights relevant for AI technologies in European health systems.

Our search resulted in a total of 70,761 documents from national repositories (international 5005; EU 844; France 6358; Germany 2587; Italy 11,147; the Netherlands 15,064; Spain 2657; United Kingdom 1032; Norway 15,721; and Türkiye 10,346), as well as 3,838 academic search results (PubMed 49; WestLaw UK 1,389; Google Scholar 2400). Ultimately, 53 laws and international treaties were included in the synthesis. Note that the Dutch repository returned individual articles of legislation rather than complete documents, resulting in an inflated number of search hits. In line with previous studies^26,27^, an entire document was included if two separate chapters of the same document were identified using the search query. Figure 1 shows the PRISMA (Preferred Reporting Items for Systematic reviews and Meta-Analyses) flowchart of the entire data collection process. Figure 2 shows high-level details of the regulatory framework for IP of AI in health systems. An overview of country-specific details is provided in Table S6.

To effectively assess the interaction between IP and health-focused human rights frameworks in the context of AI technologies, we first map how IP and IP-related rights govern both the health datasets used to train AI technologies and the AI technologies themselves within the existing legislation. Understanding the origins, scope, and limitations of IP and IP-related rights is crucial to identifying the legal relationships that arise from such rights during Hohfeldian analysis. Once this mapping is complete, we applied the Hohfeldian analysis to compare the legal relationships set out by the IP framework with those set out by health-focused human rights to anticipate possible interactions between the legal relationships underpinning these two frameworks.

### Regulatory provisions for protecting datasets

AI technologies rely on datasets, which means that IP rules for these databases are critical. Under international conventions, such as the International Covenant on Economic Social and Cultural Rights (ICESCR; Art 12, 15)^28^, Berne Convention (Art 2, 3, 9)^29^, WIPO Copyright Treaty (Art 2, 4, 5, 8)^30^, and Agreement on Trade-Related Aspects of Intellectual Property Rights^31^ (TRIPS; Art 9, 10), the databases and datasets can be protected by copyrights, trade secrets, and sui generis database rights. Directive 96/9/EC of the EU introduces the latter (Art 7)^32^, which protects databases if a substantial investment has been made to obtain, verify, or present their content even if the structure is not original that can be continuously prolonged so long as substantial changes or updates are made to the databases, whereas the EU Trade Secrets Directive establishes trade secrets as an IP-related sui generis right (Art 2). Trade secrets refer to information not commonly known and kept secret in a particular field that has commercial value due to its secrecy and is protected by reasonable measures to maintain confidentiality. Under the TRIPS Agreement, trade secrets can only be acquired, used, or disclosed with the holder’s consent (Art 39[2])^31^. The Berne Convention’s three-step test seeks to balance IP protection with the public interest by ensuring that exceptions and limitations to IP protection can exist under strict conditions that any nationally implemented limitation must comply with (Art 9[2])^29^. In other words, its goal of the three-step test is to prevent excessive limitations and respect legitimate author interests. In the EU, Directive 96/9/EC (Art 6, 9)^32^, the Data Act (Art 43)^33^, and the Digital Markets Act (Art 4) further refine these limits^34^. For instance, the Data Act establishes that sui generis database protection rights shall not apply when data is derived from connected products or related services covered by the legislation (Art 43), and the Digital Markets Act permits text and data mining for scientific research on lawfully accessed datasets (Art 4[1]). Consequently, AI technologies developed for non-commercial scientific research may still access certain datasets.

Because health data are often personal and sensitive, the General Data Protection Regulation adds another layer of rules, classifying them as a special data category requiring processing conditions (Art 4, 9)^35^. The Regulation on the European Health Data Space requires 17 categories of electronic health data to be, in principle, made available for both commercial and non-commercial research, including AI development, even if the datasets are protected by IP or IP-related rights (Art 33)^36^. Additionally, the Clinical Trials (Art 81), Medical Devices Regulation (Art 109), and In-Vitro Diagnostic Medical Devices Regulation (Art 102) permit disclosure of commercially sensitive information if it serves the public interest^37–39^. No additional provisions were found in the studied EU Member States, the United Kingdom, Norway, and Türkiye.

### Regulatory provisions for protecting health-related AI technologies

AI technologies in the health sector can be protected through various IP and IP-related rights, such as patents, trade secrets, or copyrights. Of these, only patents require official registration in Europe, whereas trade secrets and copyrights are unregistered forms of IP protection. The AI Act (Art 78[1]) and Medical Devices Regulation (Art 109[1]), and In-Vitro Diagnostic Devices Regulation (Art 102[1]) require all involved parties to maintain confidentiality and respect IP and trade secret protections^38–40^. The EU’s Directive 2016/943 allows exceptions to trade secrets protection. One such exception is a ‘legitimate interest recognised by Union or national law’ (Art 5 d); although what qualifies it remains open to court interpretations^41^.

Computer programmes as such, which form the computational foundation for AI technologies^42^, can be protected with trade secrets or copyrights^30,31^. However, the WIPO Copyright Treaty stipulates that methods of operation or mathematical concepts are not eligible for copyright protection^30^. The European Patent Convention clarifies that methods of operation or mathematical concepts cannot be patented “as such” unless they are applied to a field of technology or adapted to a specific technical implementation (Art 52), which should then be adequately disclosed^43^. Equally, the TRIPS Agreement permits the exclusion of diagnostic, therapeutic, and surgical methods for the treatment of humans or animals from patentability (Art 27[3][a]). The European Patent Convention establishes that methods of treatment by surgery and therapies are also not patentable “as such”, though diagnostics performed ex-vivo are patent-eligible (Art 53[c]). The TRIPS Agreement further acknowledges that certain licensing practices can impede competition, permitting countries to define what conditions of licensing constitute an abuse of IP or IP-related rights that has an adverse effect on competition and the market (Art 40)^31^. It also introduces the possibility of compulsory licensing, which allows governments to authorize the non-exclusive domestic use and export of a patented invention for a limited duration without the patent holder’s consent under certain circumstances. That said, the 2001 Doha Declaration clarified that compulsory licenses could be issued not only for predominantly domestic use but also to allow the export of medicines to countries lacking sufficient pharmaceutical manufacturing capacity^44^. While the Doha Declaration only relates to pharmaceutical patents, general compulsory licenses can be issued with regard to any relevant patent, seeing as patents are inherently technology-agnostic. Within national legislation, no additional or deviating IP provisions were identified in the EU Member States, the United Kingdom, Türkiye, or Norway.

Art 5(2) and 5(3) of the EU Directive 2001/29/EC provides an optional list with exceptions which permit the use of copyright-protected work without the authorisation of the rights holder, such as use of a work for teaching or scientific research or for the public security^45^. Under the AI Act, providers of general-purpose AI models must provide sufficient information about a model’s capabilities and limitations for others to use it effectively without revealing protected information and list technical specifications and licenses (Art 53)^40^. Equally, potentially copyrighted content used to train general-purpose AI models must be disclosed under the AI Act^40^. Developers of high-risk AI must demonstrate compliance with both the AI Act (Art 13) and the relevant medical device regulations^38,39^, providing authorities with the required documentation.

### Hohfeldian analysis of legal relationships

We performed a Hohfeldian analysis, which is a legal analysis technique that helps clarify the structure of legal rights and duties between different parties^46^. This analysis was applied to understand the interaction between IP rights and health-focused human rights in relation to AI technologies in the healthcare sector. The current IP framework can incentivise investment in innovation by ensuring that IP right holders have the option to enjoy the financial benefits through control over the distribution of their AI technologies in the healthcare market, while also offering governance mechanisms that seek to prevent the misuse of IP rights. When assessing the interactions between the mapped IP framework and the human rights established in the ICESCR, ICCPR, ECHR, and FCAI^28,47–49^, several identified legal relationships can create tension (see Table 1). These binding human rights instruments grant citizens the rights to self-determination and information integrity, to freely pursue their economic, social, and cultural development, and to enjoy the benefits of scientific progress and access to medical services and devices for health protection and disease prevention. At the same time, citizens have the right to not be subjected without their free consent to medical or scientific experimentation.

**Table 1:** Hohfeldian relationships established by IP legislation and health-focused human rights in the context of artificial intelligence

Legal right	Correlative obligation	Legal relationship
***Intellectual property rights***		
IP or IP-related right holder has the (time-limited) exclusive right to control or manage IP-protected intellectual creation following the mechanisms of their specific protections (Art 9, Berne Convention for copyrights; Art 27, 28, Berne Convention for patents; Art 39, Berne Convention for trade secrets; Art 7, EU Directive 96/9/EC for database rights)^29,32^.	IP or IP-related right holders have an exclusive claim on the protected AI technologies. This claim right means anyone other than the IP or IP-related right holder owes the IP or IP-related right holder a duty to respect that exclusive claim in all its provisions – unless they have acquired a appropriate permissions or unless limitations or exceptions to the claim apply.	Claim-duty
	IP or IP-related right holders have the power to grant other parties the appropriate permissions set out in the IP or IP-related right. Anyone other than the IP or IP-related right holders is subject to that exercise of power, meaning they are liable for unlawful access and use of the IP or IP-related protected materials that fall outside the permissions given by the power of the developers.	Power-liability
IP or IP-related right holders have the right to benefit materially and morally from their innovations (Art 15, ICESCR)^28^.	IP or IP-related right holders have a claim on appropriate protections that ensures that they can exercise their right to benefit from their innovations. Governments owe the IP or IP-related right holder a duty to create an environment where these rights can be exercised.	Claim-duty
IP or IP-related right holders have the right to free pursue their economic, social, and cultural development (Art 1, ICCPR)^49^.	Anyone other than the IP or IP-related holders (i.e., natural and legal persons) has no right to unlawfully infringe on the ability of IP or IP-related right holders to benefit from their innovations.	Privilege-no right
	IP or IP-related holders have the power to grant other parties the permission to benefit materially and morally from their intellectual creations. Anyone other than the IP or IP-related right holder is dependent on being granted these permissions before benefitting from the IP or IP-related protected materials.	Power-liability
IP-related right holders have the right to protect trade secrets to safeguard commercial interests (Art 39, TRIPS Agreement)^31^.	Anyone other than the IP-related right holders has no right to unlawfully acquiring, disclosing, and using trade secrets (e.g., datasets, code, software) that are not within their control. Acquiring, disclosing, and using trade secrets through lawful means (e.g., after being granted access by the IP-related right holder or by independently reverse engineering the information) is permitted	Privilege-no right
	IP-related right holders have the power to grant other parties permission to access trade secrets. Anyone other than the IP-related holder is dependent on being granted these permissions before accessing trade secrets – unless they are independently reverse-engineered.	Power-liability
***Health-focused human rights***		
Citizens and patients (i.e., natural persons) have the right to enjoy the benefits of scientific progress and its applications (Art 15b, ICESCR)^28^.	Governments owe the right holder a duty to enjoy the benefits of AI technologies in health systems and protect individuals from potential harm.	Claim-duty
	Governments are disabled from unilaterally undermining citizens’ protected right without due process or lawful justification.	Immunity-disability
Citizens and patients (i.e., natural persons) have a right to an equal and no discriminatory access to basic preventive, curative, rehabilitative health services (Art. 12.2(d), ICESCR; Art 2, ICCPR; Art 14, ECHR; Art 10, FCAI)^28,47–49^.	Governments have a duty to ensure equal and non-discriminatory access to AI-enabled healthcare services. They must refrain from discriminatory practices, and establish and enforce policies and practices guaranteeing equality in AI-enabled health service provision.	Claim-duty
	Governments have no right to prevent or unjustly hinder citizens’ exercise of this privilege, provided citizens comply with lawful conditions of service access.	Privilege-no right
	Governments are disabled from unilaterally undermining citizens’ protected rights without due process or lawful justification.	Immunity-disability
Citizens and patients (i.e., natural persons) have a right to health services that are respectful of medical ethics, culturally appropriate, designed to protect confidentiality, and improve the health status of those concerned (Art. 12.2 (d), ICESCR)^28^.	Governments owe the rights holder a duty to develop, deliver, and regulate AI technologies in the healthcare sector according to medical ethics, cultural appropriateness, confidentiality standards, and effectiveness in health improvement.	Claim-duty
	Governments have no right to unjustifiably obstruct or compromise the citizen’s use of these AI-enabled healthcare services.	Privilege-no right
	Governments are disabled from changing the legal position of citizens around receiving health services that are respectful of medical ethics, culturally appropriate, designed to protect confidentiality, and improve the health status of those concerned.	Immunity-disability
Citizens and patients (i.e., natural persons) have a right to access scientifically and medically appropriate medical services and attention of good quality in the event of sickness and to protect their health (Art 12.2(d), ICESCR)^28^.	Governments have a duty to ensure the availability, confidentiality, and accessibility (e.g., physical access, equitable, and non-discriminatory) of approved AI medical services and attention, especially for those in need of medical care or preventive health measures.	Claim-duty
	Governments have no right to interfere with citizens’ access to approved AI medical services and attention, especially for those in need of medical care or preventive health.	Privilege-no right
	Governments are disabled from unilaterally undermining citizens’ protected right without due process or lawful justification.	Immunity-disability
Citizens have the right to information about medical treatment and one’s body (Art 12, ICESCR)^28^.	Governments owe the rights holder a duty to provide citizens with clear, accurate, and accessible information about their medical treatments and the state of their health.	Claim-duty
Citizens have the right to not be subjected without their free consent to medical or scientific experimentation (Art 7, ICCPR; Art 13, FCAI)^48,49^	Governments have no right to interfere with citizens receiving clear, accurate, and accessible information about their medical treatments and the state of their health. They also have no right to interfere with individuals’ privilege to be informed about their bodies and any interventions or procedures affecting them.	Privilege-no right
	Governments are disabled from changing the legal position of citizens around receiving clear, accurate, and accessible information about their medical treatments and the state of their health.	Immunity-disability
Citizens have the right to human dignity, autonomy, self-determination, personal and bodily integrity, and information integrity (Art 1, ICESCR; Art 1, ICCPR; Art 3, Art 8, ECHR; Art 7-9 FCAI)^28,47–49^	Governments owe the rights holder a duty not to interfere with these rights without proper justification or consent. In case of interference, they have a duty to provide proper justification and obtain citizen consent.	Claim-duty
	Governments have no right to interfere with citizens’ human dignity, autonomy, self-determination, and right to integrity.	Privilege-no right
	Governments are disabled from changing the legal position of citizens surrounding human dignity, autonomy, self-determination, personal and bodily integrity, and information integrity.	Immunity-disability
Citizens have the right to a system that prevents, treats, and controls infectious diseases (Art 12.2 (c), ICESCR)^28^.	Governments owe the rights holder a duty to develop, regulate, and implement public health measures, provide necessary treatments and services, and maintain systems for disease control and prevention.	Claim-duty
	Governments have no right to arbitrarily obstruct citizens’ access or enjoyment of such public health systems.	Privilege-no right
	Governments are disabled from diminishing or eliminating these health protections without legitimate justification or procedural fairness.	Immunity-disability

*ICESCR* International Covenant for Economic, Social, and Cultural Rights, *ICCPR* International Covenant on Cultural and Political Rights, *ECHR* European Convention for Human Rights, *AI* artificial intelligence, *FCAI* Framework Convention on Artificial Intelligence and Human Rights, Democracy, and the Rule of Law.

From the Hohfeldian analysis, it becomes clear that – while the legal frameworks of IP and human rights can coexist in harmony - the applications of these frameworks for healthcare innovation must reach a balance between protecting IP right holders’ economic rights as set out in the IP law framework and citizens’ human rights to access medical services and medical attention in the event of sickness. Our analysis further showcases that health systems are tasked with balancing the right to information about medical treatment with the protection of sensitive information and ensuring commercial viability. For example, in the context of AI technologies, details on the training and development process might on one hand, help end-users understand the risks and benefits of the AI technology beyond what is captured during current regulatory and health technology assessment processes. However, such details could also affect the market position of the IP or IP-related right holder if such details are protected as trade secrets and not captured by mandatory disclosure provisions (e.g., under the AI Act, Medical Devices Regulation, Clinical Trials Regulation, or patent disclosure requirements).

Within this complex ecosystem, IP and IP-related rights are used to protect the intellectual, economic, and scientific interests of IP rights holders through different mechanisms^50^. For example, patents require public disclosure of the patented technology in exchange for its exclusive use for a specified timeframe, whereas trade secrets grant protection through non-disclosure. To ensure that IP and IP-related rights are not misused, certain limitations to these rights have been established into EU law as a way to enable access to IP-protected subject matters in limited cases of ‘legitimate interests’, and possible against remuneration in case of patents. However, any determination on what constitutes such cases is deferred to the EU and national courts^51^. The implementation of the AI Act has not fully resolved these tensions. Under the AI Act (Art 53; Annex II), providers of general-purpose AI must provide documentation in order to transparently illustrate the capabilities and limitations of the technology to end-users. For high-risk AI technologies in healthcare, developers must submit more comprehensive documentation to competent authorities, including details about internal processes and training data, to demonstrate compliance with the provisions of the AI Act (Art 13).

## Discussion

This article explores how IP legislation applies to training datasets and AI technologies in health systems, juxtaposing the rights contained in the mapped IP legislation with health-focused human rights to identify potential conflicts. Our results highlight how AI technologies, and the training data that is used to develop them, can be subject to IP protection. Notably, a distinct difference between the use of trade secrets and copyright protection and the use of patent protection is identified. Information being kept secret and that information having commercial value due to secrecy are two important criteria for earning trade secret protection (Art 39[2] of the TRIPS Agreement), as trade secrets are not publicly disclosed and must be kept secret permanently. Because of this, they can be used to protect process details that not covered by the patent scope and that are difficult to reverse-engineer. This also means that a trade secret holder cannot prevent other parties from using independently acquired technical or commercial information to generate the same information. The ability to defend and enforce their rights is, therefore, limited for trade secret owners^52^. In contrast, copyrighted materials do not have the same inherent secrecy mechanism as they may be made available to the public by the author of the work (Art 8 of the WIPO Copyright Act). Hence, while a work can be covered by copyright protections, its ideas are not (Art 9[2] of the TRIPS Agreement). In the context of AI technologies, the source code can be considered the work that is eligible for IP protection. A patent makes information public in return for the exclusive right to the invention. Although other legislation (e.g. EU AI Act [Art 13, 53], European Health Data Space Regulation [Art 33a], or Data Governance Act [Art 5]) have introduced areas where the exercise of IP rights and IP-related rights is limited by transparency and reporting requirement, the exact scope of these requirements is largely left to the interpretation of the EU and national courts^41^.

The current European IP law system offers an array of IP protection mechanisms for health datasets and AI technologies, as shown in Fig. 2. However, it is worth noting that these characteristics may only be able to partially elucidate the inner workings of AI technologies due to their black box characteristics^2,53^. Black boxes in this context comprise algorithms that no human or group of humans can closely examine to determine their inner states or processes, or can offer explanations as to how outputs were produced following the inputs available^43,54^. For instance, AI technologies can consist of billions of parameters or offer no clear logic model or rationalisation on how the outcomes are predicted^54^. Moreover, black box AI technologies could risk infringing upon the right not to be subjected to a decision based solely on automatic processing (Art 22 of the General Data Protection Regulation)^55^, although this risk should be limited for medical and public health decision-making as AI systems designated as ‘high risk’ must be able to be overseen by natural persons in European health systems (Art 14 of the AI Act)^13^. However, the intended purpose of the manufacturer forms is the essential factor in assessing whether a product qualifies as a medical device under the current interpretation of the Medical Device Regulation^56^. In other words, as long as a software product is intended for general or lifestyle and well-being purposes by the manufacturer, it could fall outside the scrutiny of the Medical Device Regulation, even if it is used in a health context. This problem is perpetuated in the AI Act, as the risk classifications between the two regulations are linked^56,57^, despite their distinctive natures; thus failing to address key problems in terms of contextual bias while only focusing on technical biases^57^. That is, the current legislative framework is mostly geared towards regulating the use and deployment of AI technologies in the EU market^13^.

More broadly, the findings highlight some difficulties in balancing the application of IP and health-focused human rights. Whereas IP rights are more geared towards realising the right to enjoy the financial benefits of one’s creation and to achieve a return of investment, health-focused human rights focus more on the realisation of the rights to enjoy the highest attainable level of health and the benefits of scientific advancement. These rights compete within health systems as new for-profit actors enter the market^23,58^. This has resulted in ethical concerns regarding the availability of essential medicine^59^. At the same time, the development and deployment of new medicines and medical technologies are incentivised and safeguarded by the prospect of IP protections that can ensure returns on investments. In the case of patents, specifically, they also direct and channel research efforts by making it possible to map and navigate the innovation landscape, while offering tech transfer instruments to enable the further deployment of the patented innovation. In the context of the collection of health data from underrepresented communities or settings, patents could become a pivotal factor for data aggregation^60^. Previously, the data aggregation power of patents was observed in the context of BRCA1 and BRCA2 genetic testing, which resulted in the patent holder accumulating extensive data about genetic variants linked to breast and ovarian cancers. Without patents, these data were likely to be scattered among many smaller entities, each holding small amounts of incomplete, low-quality data^60^.

Previous research has shown that the underrepresentation of minority populations in health datasets can bias the development of AI technologies to their detriment^17,19^. The introduction of text and data mining exceptions to IP protection in the Digital Markets Act can potentially magnify this problem because datasets covered by IP protection in the EU can become more easily accessible for non-commercial scientific research on AI technologies. This provision inherently favours population groups whose data are collected in abundance, making AI technologies easier to develop for populations with high-density data^17^. Accordingly, discrepancies in data representation are not only a matter of differences in the volume and quality of data available per population group, but also of that data being accessible for AI training purposes. This problem is not limited to the availability or accessibility of personal health data. With the emergence of ‘generative’ AI technologies, training datasets can comprise a wide variety of data types (e.g., medical textbooks, images, or audio of patient consultations), some of which may be covered by IP protection. Previous work has described the biases present in medical education and physician behaviour^61–63^, which risk becoming entrenched in AI technology when used as training data^16^.

Furthermore, the extant IP legislative framework, supported by recent UK and EU case law^64,65^, makes it clear that where the AI technology is designated as the sole inventor, outputs of AI technologies cannot (yet) receive patent protection^66^. In contrast, where AI technologies are used by natural persons as tools, IP or IP-related rights may be claimable. For instance, when a synthetic dataset with the same variables as the original dataset is generated by AI technology, the end-user of the AI technology may be able to claim sui generis database rights for the newly created dataset on the condition that any IP licence provisions of the original dataset are not infringed upon^67^. In this example, the end-user would not be able to claim traditional copyright protection for the synthetic dataset, as its structure is not their own intellectual creation – only potentially sui generis database rights. This holds important ramifications for the outputs of AI technologies that are not datasets, as it is only when AI technologies are positioned as a tool within the policy discourse, rather than as an autonomous agent, that a pathway to IP protection can open up. In other words, IP protection is potentially applicable only when natural persons remain responsible for the creative process. As a result, several critical transparency requirements for health systems have become clear: (1) AI developers need to be transparent in terms of what training datasets were used to create an AI technology and their respective IP licence characteristics^68^; (2) AI technologies need to offer insights to end-users on the (components of) training datasets that were used to produce a particular output^9,69^; and (3) health data holders need to be explicit in what IP protection is applicable to their datasets. This is also where potential conflicts with IP become critical and where regulatory provisions need to limit the scope of IP protections under certain, well-defined conditions. These regulatory requirements can be augmented with appropriate regulatory incentives (e.g., compensations, expedited regulatory approvals) to ensure that the AI innovation ecosystem services the innovators as well as the end-users.

Previous research has analysed the importance of perceptions and trust in the implementation process of AI technologies in health^70–73^. This interpretation of trust is generally described in the context of citizens, patients, and professionals due to doctor-patient relationships and is a product of transparency and openness^21,22,74^. Simultaneously, protecting the intellectual and financial investments of developers and ensuring that nobody can use their creations without their approval can be equally described as a form of building trust. In other words, the concept and importance of trust are important to consider on the side of citizens, patients, and professionals, as well as AI developers. Sufficient levels of trust are required among all stakeholder groups to incentivise the uptake and use of AI technologies among citizens, patients, and professionals, in addition to the creation of AI technologies by developers^22,75^. That said, the factors underpinning trust generation are opposing forces: where one party requires transparency, the other needs their competitive information safeguarded, while also factoring in the importance of health data protection in the context of personal and sensitive data. This suggests that a delicate balance in health systems has yet to be achieved. However, the current level of AI adoption may indicate that transparency is underprioritised in favour of confidentiality^8,76,77^. Within the EU legislation, the AI Act seeks to partially address this balance for general-purpose AI technologies by requiring general descriptions of their functionalities to be made available in such a way that they do not infringe upon IP rights. For high-risk AI technologies (e.g. AI medical devices), the requirements for transparent information for deployers of AI systems are included in the AI Act^40^.

This study has some limitations. The selection of countries was based on convenience sampling, meaning that the findings need to be cautiously applied to countries outside the scope of this study. Furthermore, the quality of the included records was not assessed. However, as the aim of this study was not to validate methodological rigour to ascertain confidence in data synthesis but to collect information about regulatory frameworks in different countries, the absence of a quality assessment does not considerably impact the study’s overall validity. The possibility of errors in translation or misinterpretation cannot be dismissed, although legal experts on IP rights were involved in assessing the completeness and accuracy of the findings. Furthermore, our analysis was limited to juxtaposing IP rights with health-focused human rights, meaning that the interactions between IP rights and other human rights that may indirectly impact the health sector were not included. Finally, we acknowledge that the decomposition of the rights identified in this article into their legal relationships is subject to interpretation. Despite our efforts to strengthen the robustness of this decomposition through various expert reviewing and validating the legal relationships and their descriptions, we cannot deny the possibility that other interpretations exist beyond the ones described here.

Several avenues for future research can be identified. First, there is currently a lack of a common understanding and interpretation of the concepts of ‘public interest’ and ‘legitimate interest’. These terms are recognised as valid justifications for limiting IP protection; yet, their precise scope remains subject to interpretation. Future work should attempt to clarify these concepts for health systems in order to develop a way to balance health and commercial interests sustainably and consistently. Second, when looking at the broader innovation ecosystem, it is worth noting that user-facing applications are currently characterised by low entry barriers and high product differentiation due to the widespread availability of application programming interfaces of cutting-edge AI models^2,78^. However, the computing power required to train and develop these cutting-edge AI models is largely concentrated within a select few large tech companies^2,17^. Future research should expand the scope of the current analysis with the provisions set out in competition law to further refine and illustrate the legal complexities of the current AI innovation ecosystem in health systems.

Ultimately, this study highlights the juxtaposition between IP and health-focused human rights frameworks. IP and IP-related rights are rooted in the human right to benefit from their innovations (ICESCR Art 15)^28^ and refined in a way that allows for boundaries to be implemented, such as in cases of text and data mining or non-commercial scientific research^79–82^. In contrast, the health-focused human rights framework is generally less prone to limitations^83^, as evidenced by the lack of a priori exceptions in international and national law regarding where human rights apply or the specific areas in which they may be limited. In order to foster an AI innovation ecosystem that meets the requirements of European health systems, a recalibration of the interpretations of IP provisions and the interplay between IP protections and regulatory requirements may be in order. That said, a degree of intransparency is inherent to the functioning of AI technologies, and we must ascertain that IP provisions do not become tasked with resolving an inherent technological limitation that falls well outside of their scope. Finally, it is important to note that policymaking in this area will have to grapple with the harsh reality of increasing global competition and the pursuit of digital sovereignty^84^, where IP and IP-related rights, trade secrets, state secrets, and other forms of protection will play a critical role.

## Methods

This study employed a validated methodological framework for mapping policies^85^, adapted from the foundational scoping review framework to enable the systematic screening of repositories instead of academic databases^86,87^. It has been previously applied to map laws, policies, and strategies in several disciplines, such as education^26,88^, employment^89^, digital health and AI^13,90^, and addiction^91^. The findings were reported using the PRISMA-ScR (Preferred Reporting Items for Systematic Reviews and Meta-Analyses extension for Scoping Reviews; Table S1) framework and analysed using qualitative document analysis methodology^92–94^.

### Eligibility criteria

Eight countries were included to provide an overview of the potentially diverse approaches to AI-related IP legislation in Europe: France, Germany, Italy, the Netherlands, Norway, Spain, Türkiye, and the United Kingdom. International and intergovernmental organisations (i.e., World Trade Organisation, World Intellectual Property Organisation [WIPO], and European Patent Organisation) were included because of their direct influence on all studied countries. EU legislation was also included because of its direct influence on the national policies of selected EU Member States (France, Germany, Italy, the Netherlands, and Spain). The selection of countries was based on convenience sampling^95^, although they represent a diverse mix of different political and health systems, country sizes, levels of digital maturity, and AI-driven economies (see Table S2)^96–98^. While IP laws are generally harmonised at the EU level, variations may exist in individual EU Member States, given that health system governance is a national competence in the EU^99^, warranting further investigation^100^.

To be eligible for inclusion in this study, documents had to be issued by government institutions and to possess legally binding force, imposing mandatory obligations on the actors subject to them (i.e., laws and international treaties). Laws and international treaties must be adhered to, with non-compliance potentially resulting in substantial penalties. Opinions, strategies, and guidance documents were not considered for inclusion as these are not legally binding. Only national legislation was considered, whereas legislations enacted by state governments in federal systems or regional and devolved governments were excluded. Documents published from 1883 onwards were considered eligible for publication, as this marks the ratification of the Paris Convention for the Protection of Industrial Property and the Berne Convention for the Protection of Literary and Artistic Works, which established the initial framework for modern IP rights^29,101^. As this study focused on understanding the legislative framework currently in effect, only active legislation was included, as well as only the latest version of the legislation. Finally, in addition to AI-specific legislation, we searched for overarching legislation governing the application of IP to computer programs, software, and health innovations.

### Data collection

Following the established methods used in policy mapping^26,27^, the data collection process consisted of five steps. First, international, European, and national repositories were searched as primary sources for data collection (see Table S3). To develop search strings for these repositories, key terms were identified, and search terms were formulated accordingly. In this study, the key terms for the AI development cycle were ‘artificial intelligence’, ‘machine learning’, ‘algorithm’, ‘data’, and ‘dataset’. The key terms for IP legislation were ‘intellectual property’, ‘copyright’, ‘patent’, ‘database rights’, and ‘trade secrets’. Data collection was performed by three authors (RVK, JS, and SF). Prior to the search, all keywords were translated into the official languages of the countries studied (see Table S4). When the combination of search terms yielded insufficient or no results, the key terms were used separately.

Second, to identify overarching political reforms or trends, supplementary searches were performed in WestLaw UK (Thomson Reuters; legal database), PubMed (health-focused database), and Google Scholar (first 300 hits per search as per previous methodological guidance)^102^. The build-up of the search string for the supplemental scientific literature search is shown in Table S5. Because of its supplementary nature, we only included the terms for AI and the selected countries, both of which were adopted to fit the scientific database. Third, legal documents and academic publications were merged to check whether they met eligibility criteria. The fourth step involved checking the reference lists for additional legal documents that might not have been identified in the search. Finally, all identified legal documents were consolidated into a single table capturing the country, legal name, year of enactment, last year of modification (if available), and relevant paragraphs from the included policies. The search in national repositories was conducted on 21 July 2024 and updated on 24 November 2024. To ensure the accuracy of the legal interpretations, we consulted three legal experts specialising in IP rights (TM; as well as experts in the Acknowledgements section).

### Data analysis

The included documents were analysed in two ways. First, a qualitative document analysis was performed to extract passages relevant to the regulation of AI^94^. To identify and map recurring themes, a deductive content analysis was conducted^103,104^. Relevant legal contents were extracted by three authors (RVK, JS, and SF) and clustered into two categories: IP legislation on training datasets and on AI technologies. Individual country information was tabulated per category, and cross-countries differences were narratively synthesised.

Second, we performed a Hohfeldian analysis, which is a legal analysis technique that helps clarify the structure of legal rights and duties between different parties^46^. The analysis seeks to categorize fundamental legal relations between parties. Hohfeld identified four pairs of correlative concepts: right-duty, privilege-no right, power-liability, and immunity-disability, each representing a distinct legal relationship. In Hohfeldian terms, the *right-duty* relationship describes the actions to which a right-holder is entitled, and how non-right-holders should act as a result to respect that entitlement. The *privilege-no right* relationship describes the actions the privilege-holder is free to do or not do without owing a duty to another party, thus outlining the actions that another party cannot restrict the privilege-holder from performing. The *power-liability* relationship captures the ability of the power-holder to alter one’s own or another’s legal rights, duties, or other legal relations, whereas the liability-holder is responsible for acting according to these changes in legal relationships. The *immunity-disability* relationship refers to the impossibility for the legal position of an immunity-holder to change regarding the topic of immunity, meaning that other parties are disabled from changing that legal position. Hohfeld’s framework aims to helps avoid confusion over the word “right,” which can mean different things in different contexts.[2] In this article, we exclusively focused on the legal concepts of right-duty and privilege-no right in the context of AI technologies, human rights, and IP rights. In this study, the correlative obligations identified through the Hohfeldian analysis of IP legislation were compared to those of health-focused human rights enshrined in international and EU legislation. This comparison aimed to identify and anticipate potential interactions, particularly in relation to the ICESCR, ICCPR, ECHR, and FCAI^28,47,48^, seeing as these are legally binding international treaties that establish the European human rights framework in the context of AI technologies.

## Supplementary information

Supplementary information

## Data Availability

All documents included in the synthesis of this study are publicly available from their respective national repositories. No new datasets were generated. As all data were publicly available and already in force in the respective countries studied, it was not necessary to request consent.
