# Supplementary material for: Artificial intelligence, intellectual property, and human rights: mapping the legal landscape in European health systems
Source: Npj Health Syst. 2025 Nov 25;2:43. doi: 10.1038/s44401-025-00050-3 (PMC13354152; doi:10.1038/s44401-025-00050-3)
Supplement: Supplementary file 1 — Supplementary information [file 44401_2025_50_MOESM1_ESM.pdf]

Supplementary Materials

van Kessel, R., Schmidt, J., van Kolfschooten, H., Feudo, S., Young, K., Valtere, L., Minssen, T., Mossialos, E. Artificial Intelligence, Intellectual Property, and Human Rights: Mapping the Legal Landscape in European Health Systems

Table of Contents

Table S1. PRISMA-ScR Reporting Checklist. 3

Table S2. Population, GDP, health expenditure, and AI investment data by studied country. 5

Table S3. A list of policy repositories used per studied country or region.. 6

Table S4. Translations of the English keywords for the policy repositories of the studied countries.. 7

Table S5. Build-up of the search strings for PubMed, WestLaw UK, and Google Scholar.................. 8

Table S6. Data extraction per included policy. 10

Table S1. PRISMA-ScR Reporting Checklist.

SECTION	ITEM	PRISMA-ScR CHECKLIST ITEM	REPORTED ON PAGE #
TITLE			
Title	1	Identify the report as a scoping review.	n/a
ABSTRACT			
Structured summary	2	Provide a structured summary that includes (as applicable): background, objectives, eligibility criteria, sources of evidence, charting methods, results, and conclusions that relate to the review questions and objectives.	2
INTRODUCTION			
Rationale	3	Describe the rationale for the review in the context of what is already known. Explain why the review questions/objectives lend themselves to a scoping review approach.	3-4
Objectives	4	Provide an explicit statement of the questions and objectives being addressed with reference to their key elements (e.g., population or participants, concepts, and context) or other relevant key elements used to conceptualize the review questions and/or objectives.	4
METHODS			
Protocol and registration	5	Indicate whether a review protocol exists; state if and where it can be accessed (e.g., a Web address); and if available, provide registration information, including the registration number.	n/a
Eligibility criteria	6	Specify characteristics of the sources of evidence used as eligibility criteria (e.g., years considered, language, and publication status), and provide a rationale.	4-5
Information sources*	7	Describe all information sources in the search (e.g., databases with dates of coverage and contact with authors to identify additional sources), as well as the date the most recent search was executed.	5-6 & Table S2 in appendix
Search	8	Present the full electronic search strategy for at least 1 database, including any limits used, such that it could be repeated.	5-6 & Table S3 in appendix
Selection of sources of evidence†	9	State the process for selecting sources of evidence (i.e., screening and eligibility) included in the scoping review.	5-6
Data charting process‡	10	Describe the methods of charting data from the included sources of evidence (e.g., calibrated forms or forms that have been tested by the team before their use, and whether data charting was done independently or in duplicate) and any processes for obtaining and confirming data from investigators.	5
Data items	11	List and define all variables for which data were sought and any assumptions and simplifications made.	n/a
Critical appraisal of individual sources of evidence§	12	If done, provide a rationale for conducting a critical appraisal of included sources of evidence; describe the methods used and how this information was used in any data synthesis (if appropriate).	n/a

Synthesis of results	13	Describe the methods of handling and summarizing the data that were charted.	6
RESULTS			
Selection of sources of evidence	14	Give numbers of sources of evidence screened, assessed for eligibility, and included in the review, with reasons for exclusions at each stage, ideally using a flow diagram.	6-7
Characteristics of sources of evidence	15	For each source of evidence, present characteristics for which data were charted and provide the citations.	Table S5 in the appendix
Critical appraisal within sources of evidence	16	If done, present data on critical appraisal of included sources of evidence (see item 12).	n/a
Results of individual sources of evidence	17	For each included source of evidence, present the relevant data that were charted that relate to the review questions and objectives.	eTables 5 in appendix
Synthesis of results	18	Summarize and/or present the charting results as they relate to the review questions and objectives.	8-12
DISCUSSION			
Summary of evidence	19	Summarize the main results (including an overview of concepts, themes, and types of evidence available), link to the review questions and objectives, and consider the relevance to key groups.	12-14
Limitations	20	Discuss the limitations of the scoping review process.	14-15
Conclusions	21	Provide a general interpretation of the results with respect to the review questions and objectives, as well as potential implications and/or next steps.	15
FUNDING			
Funding	22	Describe sources of funding for the included sources of evidence, as well as sources of funding for the scoping review. Describe the role of the funders of the scoping review.	15

Table S2. Population, GDP, health expenditure, and AI investment data by studied country.

Country	Population (2023; million)	Gross Domestic Product, PPP (2023; USD trillion)	Current health expenditure (2021; % of GDP)	Current health expenditure per capita, PPP (2021; USD)	Venture capital investment in AI by 2023 (Cumulative; USD million)	Number of investments by 2023 (Cumulative)
European Union-27	449,48	18,35	11	5226,5	50873	4624
France	68,17	3,03	12,3	6330,5	11033	1014
Germany	84,48	4,46	12,9	7607	16352	1130
Italy	58,76	2,25	9,4	4372	990	187
Netherlands	17,87	1,12	11,3	7179	1447	237
Norway	5,52	0,49	10,1	8275	1194	130
Spain	48,37	1,58	10,7	4367,6	3889	520
Turkiye	85,33	1,11	4,6	1390	2412	64
United Kingdom	68,35	3,34	12,4	6159,8	29871	2651
	Data was obtained from the World Bank. Sources: World Bank Database (https://data.worldbank.org/) [accessed 20 November 2024].				Data was obtained from the OECD. Sources: OECD.AI Policy Observatory Live Data (https://oecd.ai/en/data) [accessed 20 November 2024	

Table S3. A list of policy repositories used per studied country or region.

**Country**	**Policy repository**
United Nations	https://search.un.org
World Intellectual Property Organization	https://www.wipo.int/en/web/wipolex/index [search database]<br>https://www.wipo.int/treaties/en/<br>[manual search of WIPO treaties]
World Trade Organization	https://docs.wto.org
European Patent Office	https://www.epo.org/en [using search function, filtering for Legal texts]
European Union	https://eur-lex.europa.eu
France	https://www.legifrance.gouv.fr/
Germany	https://www.bgbl.de/xaver/bgbl/start<br>https://www.recht.bund.de/de/home/home_node.html#research [filters: "Gesetze", "Verordnung"]
Italy	https://www.normattiva.it/
Netherlands	https://www.inview.nl
Norway	https://lovdata.no/
Spain	http://boe.es/
Türkiye	https://www.resmigazete.gov.tr/
United Kingdom	https://legislation.gov.uk

Table S4. Translations of the English keywords for the policy repositories of the studied countries.

English	French	Italian	Dutch	German	Spanish	Norwegian	Turkish
Artificial intelligence	Intelligence artificielle	Intelligenza artificiale	Kunstmatige intelligentie	Künstliche Intelligenz	Inteligencia artificial	Kunstig intelligens	Yapay zeka
Machine learning	apprentissage automatique	apprendimento automatico	machinaal leren	maschinelles Lernen	aprendizaje automático	Maskinlæring	Makine öğrenimi
Algorithm	algorithme	algoritmo	algoritme	Algorithmus	algoritmo	algoritme	algoritma
Data	données	dati	gegevens	Daten	datos	data	Veri
Dataset	ensemble de données	set di dati	dataset	Datensatz	conjunto de datos	datasett	veri kümesi
Intellectual property	propriété intellectuelle	proprietà intellettuale	intellectueel eigendom	geistiges Eigentum	propiedad intelectual	intellektuell eiendom	fikri mülkiyet
Copyright	droit d'auteur	copyright	auteursrecht	Urheberrecht	derechos de autor	opphavsrett	telif hakkı
Patent	brevet	brevetto	octrooi	Patent	patente	patent	patent
Trade secret	secret commercial	segreto commerciale	handelsgeheim	Geschäftsgeheimni s	secreto comercial	forretningshemme lighet	ticari sır

Table S5. Build-up of the search strings for PubMed, WestLaw UK, and Google Scholar.

Database	Search query	String
PubMed	#1	"Artificial Intelligence"[MeSH] OR Algorithm[MeSH] OR "Machine Learning"[MeSH] OR "Deep Learning"[MeSH] OR "Expert Systems"[MeSH] OR "Natural Language Processing"[MeSH] OR AI[Title/Abstract] OR "artificial intelligence"[Title/Abstract] OR "Machine Learning"[Title/Abstract] OR "Deep learning"[Title/Abstract] OR "neural network*"[Title/Abstract] OR "supervised learning"[Title/Abstract] OR "unsupervised learning"[Title/Abstract]
	#2	"intellectual property"[Title/Abstract] OR copyright[Title/Abstract] OR patent[Title/Abstract] OR "trade secret*"[Title/Abstract]
	#3	"United Kingdom"[Title/Abstract] OR "Engl*"[Title/Abstract] OR Wales[Title/Abstract] OR Welsh OR Scot*[Title/Abstract] OR "Northern Ir*"[Title/Abstract] OR German*[Title/Abstract] OR Dutch[Title/Abstract] OR Netherlands[Title/Abstract] OR Ital*[Title/Abstract] OR Spanish[Title/Abstract] OR Spain[Title/Abstract] OR France[Title/Abstract] OR French[Title/Abstract] OR Turk*[Title/Abstract] OR Norw*[Title/Abstract]
	Final	("Artificial Intelligence"[MeSH] OR Algorithm[MeSH] OR "Machine Learning"[MeSH] OR "Deep Learning"[MeSH] OR "Expert Systems"[MeSH] OR "Natural Language Processing"[MeSH] OR AI[Title/Abstract] OR "artificial intelligence"[Title/Abstract] OR "Machine Learning"[Title/Abstract] OR "Deep learning"[Title/Abstract] OR "neural network*"[Title/Abstract] OR "supervised learning"[Title/Abstract] OR "unsupervised learning"[Title/Abstract]) AND ("intellectual property"[Title/Abstract] OR copyright[Title/Abstract] OR patent[Title/Abstract] OR "trade secret*"[Title/Abstract]) AND ("United Kingdom"[Title/Abstract] OR "Engl*"[Title/Abstract] OR Wales[Title/Abstract] OR Welsh OR Scot*[Title/Abstract] OR "Northern Ir*"[Title/Abstract] OR German*[Title/Abstract] OR Dutch[Title/Abstract] OR Netherlands[Title/Abstract] OR Ital*[Title/Abstract] OR Spanish[Title/Abstract] OR Spain[Title/Abstract] OR France[Title/Abstract] OR French[Title/Abstract] OR Turk*[Title/Abstract] OR Norw*[Title/Abstract])
WestLaw UK	#1	"Artificial Intelligence" or Algorithm OR "Machine Learning" or "Deep Learning" or "Expert Systems" or "Natural Language Processing" or AI or "neural network*" or "supervised learning" or "unsupervised learning"
	#2	"intellectual property" or copyright or patent or "trade secret*"
	#3	"United Kingdom" or "Engl*" or Wales or Welsh or Scot* or "Northern Ir*" or German* or Dutch or Netherlands or Ital* or Spanish or Spain or France or French or Turk* or Norw*
	Final	("Artificial Intelligence" or Algorithm OR "Machine Learning" or "Deep Learning" or "Expert Systems" or "Natural Language Processing" or AI or "neural network*" or "supervised learning" or "unsupervised learning") & ("intellectual property" or copyright or patent or "trade secret*") & ("United Kingdom" or "Engl*" or Wales or Welsh or Scot* or "Northern Ir*" or German* or Dutch or Netherlands or Ital* or Spanish or Spain or France or French or Turk* or Norw*)

Google Scholar		"Artificial Intelligence" “intellectual property rights” “United kingdom”<br>"Artificial Intelligence" “intellectual property rights” Germany<br>"Artificial Intelligence" “intellectual property rights” Netherlands<br>"Artificial Intelligence" “intellectual property rights” Italy<br>"Artificial Intelligence" “intellectual property rights” Spain<br>"Artificial Intelligence" “intellectual property rights” France<br>"Artificial Intelligence" “intellectual property rights” Turkey<br>"Artificial Intelligence" “intellectual property rights” Norway

Table S6. Data extraction per included policy.

Institution	Year	Year updated	Original name	Content
United Nations	1966		International Covenant on Economic, Social and Cultural Rights	Art 12(1). The States Parties to the present Covenant recognize the right of everyone to the enjoyment of the highest attainable standard of physical and mental health.<br>Art 12(2). The steps to be taken by the States Parties to the present Covenant to achieve the full realization of this right shall include those necessary for:<br>(c) The prevention, treatment and control of epidemic, endemic, occupational and other diseases;<br>(d) The creation of conditions which would assure access to all medical service and medical attention in the event of sickness.<br>Art 15(1). The States Parties to the present Covenant recognize the right of everyone:<br>(b) To enjoy the benefits of scientific progress and its applications;<br>(c) To benefit from the protection of the moral and material interests resulting from any scientific, literary or artistic production of which he is the author.<br>Art 15(2). The steps to be taken by the States Parties to the present Covenant to achieve the full realization of this right shall include those necessary for the conservation, the development and the diffusion of science and culture.<br>Art 15(4). The States Parties to the present Covenant recognize the benefits to be derived from the encouragement and development of international contacts and co-operation in the scientific and cultural fields.
World Intellectual Property Organization	1883	1979	Paris Convention	Art 5(a)(2). Each country of the Union shall have the right to take legislative measures providing for the grant of compulsory licenses to prevent the abuses which might result from the exercise of the exclusive rights conferred by the patent, for example, failure to work.
World Intellectual Property Organization	1886	1979	Berne Convention for the Protection of Literary and Artistic Works	Art 2(1). The expression “literary and artistic works” shall include every production in the literary, scientific and artistic domain, whatever may be the mode or form of its expression, such as books, pamphlets and other writings; lectures, addresses, sermons and other works of the same nature; dramatic or dramatico-musical works; choreographic works and entertainments in dumb show; musical compositions with or without words; cinematographic works to which are assimilated works expressed by a process analogous to cinematography; works of drawing, painting, architecture, sculpture, engraving and lithography; photographic works to which are assimilated works expressed by a process analogous to photography; works of applied art; illustrations, maps, plans, sketches and three-dimensional works relative to geography,

				topography, architecture or science.<br>Art 3(1). The protection of this Convention shall apply to:<br>(a) authors who are nationals of one of the countries of the Union, for their works, whether published or not;<br>(b) authors who are not nationals of one of the countries of the Union, for their works first published in one of those countries, or simultaneously in a country outside the Union and in a country of the Union.<br>Art 9(1). Authors of literary and artistic works protected by this Convention shall have the exclusive right of authorizing the reproduction of these works, in any manner or form.<br>Art 9(2). It shall be a matter for legislation in the countries of the Union to permit the reproduction of such works in certain special cases, provided that such reproduction does not conflict with a normal exploitation of the work and does not unreasonably prejudice the legitimate interests of the author.
World Intellectual Property Organization	1996		WIPO Copyright Treaty	Art 2. Copyright protection extends to expressions and not to ideas, procedures, methods of operation or mathematical concepts as such.<br>Art 4. Computer programs are protected as literary works within the meaning of Article 2 of the Berne Convention. Such protection applies to computer programs, whatever may be the mode or form of their expression.<br>Art 5. Compilations of data or other material, in any form, which by reason of the selection or arrangement of their contents constitute intellectual creations, are protected as such. This protection does not extend to the data or the material itself and is without prejudice to any copyright subsisting in the data or material contained in the compilation.<br>Art 8. Without prejudice to the provisions of Articles 11(1)(ii), 11bis(1)(i) and (ii), 11ter(1)(ii), 14(1)(ii) and 14bis(1) of the Berne Convention, authors of literary and artistic works shall enjoy the exclusive right of authorizing any communication to the public of their works, by wire or wireless means, including the making available to the public of their works in such a way that members of the public may access these works from a place and at a time individually chosen by them
World Trade Organization	1995		Agreement On Trade-Related Aspects Of Intellectual Property Rights (TRIPS)	Art 7. The protection and enforcement of intellectual property rights should contribute to the promotion of technological innovation and to the transfer and dissemination of technology, to the mutual advantage of producers and users of technological knowledge and in a manner conducive to social and economic welfare, and to a balance of rights and obligations.<br>Art 8(1). Members may, in formulating or amending their laws and regulations, adopt measures necessary to protect public health and nutrition, and to promote the public

				interest in sectors of vital importance to their socio-economic and technological development, provided that such measures are consistent with the provisions of this Agreement.<br>Art 8(2). Appropriate measures, provided that they are consistent with the provisions of this Agreement, may be needed to prevent the abuse of intellectual property rights by right holders or the resort to practices which unreasonably restrain trade or adversely affect the international transfer of technology.<br>Art 9(2). Copyright protection shall extend to expressions and not to ideas, procedures, methods of operation or mathematical concepts as such.<br>Art 10(1). Computer programs, whether in source or object code, shall be protected as literary works under the Berne Convention (1971).<br>Art 10(2). Compilations of data or other material, whether in machine readable or other form, which by reason of the selection or arrangement of their contents constitute intellectual creations shall be protected as such. Such protection, which shall not extend to the data or material itself, shall be without prejudice to any copyright subsisting in the data or material itself.<br>Art 12. Whenever the term of protection of a work, other than a photographic work or a work of applied art, is calculated on a basis other than the life of a natural person, such term shall be no less than 50 years from the end of the calendar year of authorized publication, or, failing such authorized publication within 50 years from the making of the work, 50 years from the end of the calendar year of making.<br>Art 13. Members shall confine limitations or exceptions to exclusive rights to certain special cases which do not conflict with a normal exploitation of the work and do not unreasonably prejudice the legitimate interests of the right holder.<br>Art 27(1). Subject to the provisions of paragraphs 2 and 3, patents shall be available for any inventions, whether products or processes, in all fields of technology, provided that they are new, involve an inventive step and are capable of industrial application.<br>Art 27(2). Members may exclude from patentability inventions, the prevention within their territory of the commercial exploitation of which is necessary to protect ordre public or morality, including to protect human, animal or plant life or health or to avoid serious prejudice to the environment, provided that such exclusion is not made merely because the exploitation is prohibited by their law.<br>Art 27(3). Members may also exclude from patentability:<br>(a) diagnostic, therapeutic and surgical methods for the treatment of humans or animals;<br>Art 28(1). A patent shall confer on its owner the following exclusive rights:

				(a) where the subject matter of a patent is a product, to prevent third parties not having the owner's consent from the acts of: making, using, offering for sale, selling, or importing for these purposes that product;<br>(b) where the subject matter of a patent is a process, to prevent third parties not having the owner's consent from the act of using the process, and from the acts of: using, offering for sale, selling, or importing for these purposes at least the product obtained directly by that process.<br>Art 28(2). Patent owners shall also have the right to assign, or transfer by succession, the patent and to conclude licensing contracts.<br>Art 30. Members may provide limited exceptions to the exclusive rights conferred by a patent, provided that such exceptions do not unreasonably conflict with a normal exploitation of the patent and do not unreasonably prejudice the legitimate interests of the patent owner, taking account of the legitimate interests of third parties.<br>Art 31. Where the law of a Member allows for other use of the subject matter of a patent without the authorization of the right holder, including use by the government or third parties authorized by the government, the following provisions shall be respected:<br>(a) authorization of such use shall be considered on its individual merits;<br>(b) such use may only be permitted if, prior to such use, the proposed user has made efforts to obtain authorization from the right holder on reasonable commercial terms and conditions and that such efforts have not been successful within a reasonable period of time. This requirement may be waived by a Member in the case of a national emergency or other circumstances of extreme urgency or in cases of public non-commercial use. In situations of national emergency or other circumstances of extreme urgency, the right holder shall, nevertheless, be notified as soon as reasonably practicable. In the case of public non-commercial use, where the government or contractor, without making a patent search, knows or has demonstrable grounds to know that a valid patent is or will be used by or for the government, the right holder shall be informed promptly;<br>(c) the scope and duration of such use shall be limited to the purpose for which it was authorized, and in the case of semi-conductor technology shall only be for public non-commercial use or to remedy a practice determined after judicial or administrative process to be anti-competitive;<br>(d) such use shall be non-exclusive;<br>(e) such use shall be non-assignable, except with that part of the enterprise or goodwill which enjoys such use;<br>(f) any such use shall be authorized predominantly for the supply of the domestic market of the Member authorizing such use;

				(g) authorization for such use shall be liable, subject to adequate protection of the legitimate interests of the persons so authorized, to be terminated if and when the circumstances which led to it cease to exist and are unlikely to recur. The competent authority shall have the authority to review, upon motivated request, the continued existence of these circumstances;<br>(h) the right holder shall be paid adequate remuneration in the circumstances of each case, taking into account the economic value of the authorization;<br>(i) the legal validity of any decision relating to the authorization of such use shall be subject to judicial review or other independent review by a distinct higher authority in that Member;<br>(j) any decision relating to the remuneration provided in respect of such use shall be subject to judicial review or other independent review by a distinct higher authority in that Member;<br>(k) Members are not obliged to apply the conditions set forth in subparagraphs (b) and (f) where such use is permitted to remedy a practice determined after judicial or administrative process to be anti-competitive. The need to correct anti-competitive practices may be taken into account in determining the amount of remuneration in such cases. Competent authorities shall have the authority to refuse termination of authorization if and when the conditions which led to such authorization are likely to recur;<br>(l) where such use is authorized to permit the exploitation of a patent ("the second patent") which cannot be exploited without infringing another patent ("the first patent"), the following additional conditions shall apply:<br>(i) the invention claimed in the second patent shall involve an important technical advance of considerable economic significance in relation to the invention claimed in the first patent;<br>(ii) the owner of the first patent shall be entitled to a cross-licence on reasonable terms to use the invention claimed in the second patent; and<br>(iii) the use authorized in respect of the first patent shall be non-assignable except with the assignment of the second patent.<br>Art 39(2). Natural and legal persons shall have the possibility of preventing information lawfully within their control from being disclosed to, acquired by, or used by others without their consent in a manner contrary to honest commercial practices (10) so long as such information:<br>(a) is secret in the sense that it is not, as a body or in the precise configuration and assembly of its components, generally known among or readily accessible to persons within the circles that normally deal with the kind of information in question;

				(b) has commercial value because it is secret; and<br>(c) has been subject to reasonable steps under the circumstances, by the person lawfully in control of the information, to keep it secret.<br>Art 30. Members may provide limited exceptions to the exclusive rights conferred by a patent, provided that such exceptions do not unreasonably conflict with a normal exploitation of the patent and do not unreasonably prejudice the legitimate interests of the patent owner, taking account of the legitimate interests of third parties.<br>Art 39(3). Members, when requiring, as a condition of approving the marketing of pharmaceutical or of agricultural chemical products which utilize new chemical entities, the submission of undisclosed test or other data, the origination of which involves a considerable effort, shall protect such data against unfair commercial use. In addition, Members shall protect such data against disclosure, except where necessary to protect the public, or unless steps are taken to ensure that the data are protected against unfair commercial use.<br>Art 40(1). Members agree that some licensing practices or conditions pertaining to intellectual property rights which restrain competition may have adverse effects on trade and may impede the transfer and dissemination of technology.<br>Art 40(2). Nothing in this Agreement shall prevent Members from specifying in their legislation licensing practices or conditions that may in particular cases constitute an abuse of intellectual property rights having an adverse effect on competition in the relevant market. As provided above, a Member may adopt, consistently with the other provisions of this Agreement, appropriate measures to prevent or control such practices, which may include for example exclusive grantback conditions, conditions preventing challenges to validity and coercive package licensing, in the light of the relevant laws and regulations of that Member.
World Trade Organization	2001	2001	Doha Declaration on the TRIPS agreement and public health	1. We recognize the gravity of the public health problems afflicting many developing and least-developed countries, especially those resulting from HIV/AIDS, tuberculosis, malaria and other epidemics.<br>2. We stress the need for the WTO Agreement on Trade-Related Aspects of Intellectual Property Rights (TRIPS Agreement) to be part of the wider national and international action to address these problems.<br>3. We recognize that intellectual property protection is important for the development of new medicines. We also recognize the concerns about its effects on prices.<br>4. We agree that the TRIPS Agreement does not and should not prevent members from taking measures to protect public health. Accordingly, while reiterating our commitment to the TRIPS Agreement, we affirm that the Agreement can and should be interpreted and implemented in a manner supportive of WTO members' right to protect

				public health and, in particular, to promote access to medicines for all. In this connection, we reaffirm the right of WTO members to use, to the full, the provisions in the TRIPS Agreement, which provide flexibility for this purpose.<br>5. Accordingly and in the light of paragraph 4 above, while maintaining our commitments in the TRIPS Agreement, we recognize that these flexibilities include:<br>(a) In applying the customary rules of interpretation of public international law, each provision of the TRIPS Agreement shall be read in the light of the object and purpose of the Agreement as expressed, in particular, in its objectives and principles.<br>(b) Each member has the right to grant compulsory licences and the freedom to determine the grounds upon which such licences are granted.<br>(c) Each member has the right to determine what constitutes a national emergency or other circumstances of extreme urgency, it being understood that public health crises, including those relating to HIV/AIDS, tuberculosis, malaria and other epidemics, can represent a national emergency or other circumstances of extreme urgency.<br>(d) The effect of the provisions in the TRIPS Agreement that are relevant to the exhaustion of intellectual property rights is to leave each member free to establish its own regime for such exhaustion without challenge, subject to the MFN and national treatment provisions of Articles 3 and 4.<br>6. We recognize that WTO members with insufficient or no manufacturing capacities in the pharmaceutical sector could face difficulties in making effective use of compulsory licensing under the TRIPS Agreement. We instruct the Council for TRIPS to find an expeditious solution to this problem and to report to the General Council before the end of 2002.<br>7. We reaffirm the commitment of developed-country members to provide incentives to their enterprises and institutions to promote and encourage technology transfer to least-developed country members pursuant to Article 66.2. We also agree that the least-developed country members will not be obliged, with respect to pharmaceutical products, to implement or apply Sections 5 and 7 of Part II of the TRIPS Agreement or to enforce rights provided for under these Sections until 1 January 2016, without prejudice to the right of least-developed country members to seek other extensions of the transition periods as provided for in Article 66.1 of the TRIPS Agreement. We instruct the Council for TRIPS to take the necessary action to give effect to this pursuant to Article 66.1 of the TRIPS Agreement.
European Patent Office	1973	2020	European Patent Convention	Art 52(1). European patents shall be granted for any inventions, in all fields of technology, provided that they are new, involve an inventive step and are susceptible of industrial application.

				Art 52(2). The following in particular shall not be regarded as inventions within the meaning of paragraph 1:<br>(a) discoveries, scientific theories and mathematical methods;<br>(b) aesthetic creations;<br>(c) schemes, rules and methods for performing mental acts, playing games or doing business, and programs for computers;<br>(d) presentations of information.<br>Art 52(3). Paragraph 2 shall exclude the patentability of the subject-matter or activities referred to therein only to the extent to which a European patent application or European patent relates to such subject-matter or activities as such.<br>Art 53. European patents shall not be granted in respect of:<br>(a) inventions the commercial exploitation of which would be contrary to "ordre public" or morality; such exploitation shall not be deemed to be so contrary merely because it is prohibited by law or regulation in some or all of the Contracting States;<br>(b) plant or animal varieties or essentially biological processes for the production of plants or animals; this provision shall not apply to microbiological processes or the products thereof;<br>(c) methods for treatment of the human or animal body by surgery or therapy and diagnostic methods practised on the human or animal body; this provision shall not apply to products, in particular substances or compositions, for use in any of these methods.
European Patent Office	2024		Guidelines for Examination in the European Patent Office	GII-3.3. Mathematical methods play an important role in the solution of technical problems in all fields of technology. However, they are excluded from patentability under Art. 52(2)(a) when claimed as such (Art. 52(3)). The exclusion applies if a claim is directed to a purely abstract mathematical method and the claim does not require any technical means. If a claim is directed either to a method involving the use of technical means (e.g. a computer) or to a device, its subject-matter has a technical character as a whole and is thus not excluded from patentability under Art. 52(2) and (3).<br><br>Merely specifying the technical nature of the data or parameters of the mathematical method may not be sufficient on its own to define an invention within the meaning of Art. 52(1). Even if the resulting method would not be considered a purely abstract mathematical method as such within the meaning of Art. 52(2)(a) and (3), it may still fall under the excluded category of methods for performing mental acts as such if no use of technical means is implied (Art. 52(2)(c) and (3); see G-II, 3.5.1).

				Once it is established that the claimed subject-matter as a whole is not excluded from patentability under Art. 52(2) and (3) and is thus an invention within the meaning of Art. 52(1), it is examined in respect of the other requirements of patentability, in particular novelty and inventive step (G-I, 1). For the assessment of inventive step, all features which contribute to the technical character of the invention must be taken into account (G-VII, 5.4). When the claimed invention is based on a mathematical method, it is assessed whether the mathematical method contributes to the technical character of the invention. A mathematical method may contribute to the technical character of an invention, i.e. contribute to producing a technical effect that serves a technical purpose, by its application to a field of technology and/or by being adapted to a specific technical implementation (T 2330/13).<br><br>GII-3.3.1. Artificial intelligence and machine learning are based on computational models and algorithms for classification, clustering, regression and dimensionality reduction, such as neural networks, genetic algorithms, support vector machines, k-means, kernel regression and discriminant analysis. Such computational models and algorithms are per se of an abstract mathematical nature, irrespective of whether they can be "trained" based on training data. Hence, the guidance provided in G-II, 3.3 generally applies also to such computational models and algorithms.<br><br>Terms such as "support vector machine", "reasoning engine" or "neural network" may, depending on the context, merely refer to abstract models or algorithms and thus do not, on their own, necessarily imply the use of a technical means. This has to be taken into account when examining whether the claimed subject-matter has a technical character as a whole (Art. 52(1), (2) and (3)).<br><br>The technical effect that a machine learning algorithm achieves may be readily apparent or established by explanations, mathematical proof, experimental data or the like. While mere allegations are not enough, comprehensive proof is not required either. If the technical effect is dependent on particular characteristics of the training dataset used, those characteristics that are required to reproduce the technical effect must be disclosed unless the skilled person can determine them without undue burden using common general knowledge. However, in general, there is no need to disclose the specific training dataset itself.<br><br>GII-4.2.1.3. Diagnostic methods likewise do not cover all methods related to diagnosis.

				To determine whether a claim is directed to a diagnostic method within the meaning of Art. 53(c) and so excluded from patentability, it must first be established whether it includes all of the necessary phases (G 1/04).<br><br>The claim must include method steps relating to all of the following phases:<br>(i) the examination phase, involving the collection of data<br>(ii) the comparison of the data with standard values<br>(iii) the finding of any significant deviation, i.e. a symptom, during the comparison<br>(iv) the attribution of the deviation to a particular clinical picture, i.e. the deductive medical or veterinary decision phase (diagnosis for curative purposes in the strict sense).<br><br>In addition, a method is only regarded as a diagnostic method within the meaning of Art. 53(c), and so excluded from patentability, if all method steps of a technical nature belonging to the preceding steps which are constitutive for making the diagnosis, i.e. phases (i)-(iii), satisfy the criterion "practised on the human or animal body". However, the steps of phases (ii) and (iii) which consist in comparing the data collected in the examination phase with standard values and in finding a significant deviation resulting from the comparison are not subject to this criterion, because these activities are predominantly of a non-technical nature and are normally not practised on the human or animal body. This means that, in most cases, only phase (i), i.e. the examination phase involving the collection of data, can actually be of a technical nature within the meaning of G 1/04 and so concerned by the criterion "practised on the human or animal body" (see T 1197/02, T 143/04, T 1016/10).<br><br>Only the steps strictly describing phases (i)-(iv) have to be taken into account in determining the diagnostic character of the claimed method. Additional, preparatory or intermediate steps which may be introduced into the claimed method are irrelevant for this question (see T 1197/02, T 143/04, T 1016/10). For example, a claim may comprise preparatory steps of adjusting or preparing the apparatus used to collect data. However, these additional features are not part of any of phases (i)-(iii), which are constitutive for making the diagnosis. Likewise, data processing using an automated apparatus is not actually part of the examination phase, which involves collecting data, but it results from a subsequent step, intermediate between data collection and the comparison of the collected data with standard values. The issue of whether or not such additional steps are of a technical nature and practised on the human or animal

				body is therefore irrelevant for assessing whether a claimed method is a diagnostic method falling under the exception clause of Art. 53(c).<br><br>For a method step of a technical nature to meet the criterion "practised on the human or animal body", there must be an interaction with the human or animal body. The type or intensity of the interaction is not decisive: the criterion is fulfilled if the performance of the method step in question necessitates the presence of the body. Direct physical contact with the body is not required. A medical or veterinary practitioner does not have to be involved in the procedure, be it by being present or by bearing the overall responsibility.<br><br>If all of the above criteria are satisfied, then the claim defines a diagnostic method practised on the human or animal body, and an objection will be raised under Art. 53(c). Accordingly, methods for merely obtaining information (data, physical quantities) from the living human or animal body (e.g. X-ray investigations, MRI studies, and blood pressure measurements) are not excluded from patentability under Art. 53(c).
European Union	1996	2019	Directive 96/9/EC of the European Parliament and of the Council of 11 March 1996 on the legal protection of databases (1/3)	Art 1(2). For the purposes of this Directive, ‘database’ shall mean a collection of independent works, data or other materials arranged in a systematic or methodical way and individually accessible by electronic or other means.<br>Art 1(3). Protection under this Directive shall not apply to computer programs used in the making or operation of databases accessible by electronic means.<br>Art 3(1). In accordance with this Directive, databases which, by reason of the selection or arrangement of their contents, constitute the author's own intellectual creation shall be protected as such by copyright. No other criteria shall be applied to determine their eligibility for that protection.<br>Art 3(2). The copyright protection of databases provided for by this Directive shall not extend to their contents and shall be without prejudice to any rights subsisting in those contents themselves.<br>Art 4(1). The author of a database shall be the natural person or group of natural persons who created the base or, where the legislation of the Member States so permits, the legal person designated as the rightholder by that legislation.<br>Art 4(2). Where collective works are recognized by the legislation of a Member State, the economic rights shall be owned by the person holding the copyright.<br>Art 4(3). In respect of a database created by a group of natural persons jointly, the exclusive rights shall be owned jointly.

				Art 5. In respect of the expression of the database which is protectable by copyright, the author of a database shall have the exclusive right to carry out or to authorize:<br>(a) temporary or permanent reproduction by any means and in any form, in whole or in part;<br>(b) translation, adaptation, arrangement and any other alteration;<br>(c) any form of distribution to the public of the database or of copies thereof. The first sale in the Community of a copy of the database by the rightholder or with his consent shall exhaust the right to control resale of that copy within the Community;<br>(d) any communication, display or performance to the public;<br>(e) any reproduction, distribution, communication, display or performance to the public of the results of the acts referred to in (b).
European Union	1996	2019	Directive 96/9/EC of the European Parliament and of the Council of 11 March 1996 on the legal protection of databases (2/3)	Art 6(1). The performance by the lawful user of a database or of a copy thereof of any of the acts listed in Article 5 which is necessary for the purposes of access to the contents of the databases and normal use of the contents by the lawful user shall not require the authorization of the author of the database. Where the lawful user is authorized to use only part of the database, this provision shall apply only to that part.<br>Art 6(2). Member States shall have the option of providing for limitations on the rights set out in Article 5 in the following cases:<br>(b) where there is use for the sole purpose of illustration for teaching or scientific research, as long as the source is indicated and to the extent justified by the non-commercial purpose to be achieved, without prejudice to the exceptions and limitations provided for in Directive (EU) 2019/790 of the European Parliament and of the Council<br>Art 7(1). Member States shall provide for a right for the maker of a database which shows that there has been qualitatively and/or quantitatively a substantial investment in either the obtaining, verification or presentation of the contents to prevent extraction and/or re-utilization of the whole or of a substantial part, evaluated qualitatively and/or quantitatively, of the contents of that database.<br>Art 7(2). For the purposes of this Chapter:<br>(a) ‘extraction’ shall mean the permanent or temporary transfer of all or a substantial part of the contents of a database to another medium by any means or in any form;<br>(b) ‘re-utilization’ shall mean any form of making available to the public all or a substantial part of the contents of a database by the distribution of copies, by renting, by online or other forms of transmission. The first sale of a copy of a database within the Community by the rightholder or with his consent shall exhaust the right to control resale of that copy within the Community;

European Union	1996	2019	Directive 96/9/EC of the European Parliament and of the Council of 11 March 1996 on the legal protection of databases (3/3)	Art 9. Member States may stipulate that lawful users of a database which is made available to the public in whatever manner may, without the authorization of its maker, extract or re-utilize a substantial part of its contents:<br>(b) in the case of extraction for the purposes of illustration for teaching or scientific research, as long as the source is indicated and to the extent justified by the non-commercial purpose to be achieved, without prejudice to the exceptions and limitations provided for in Directive (EU) 2019/790;
European Union	2000		Charter of Fundamental Rights of the European Union	Art 17(2). Intellectual property shall be protected.
European Union	2001		Directive 2001/83/EC of the European Parliament and of the Council of 6 November 2001 on the Community code relating to medicinal products for human use	Art 1. For the purposes of this Directive, the following terms shall bear the following meanings:<br>1. Proprietary medicinal product: Any ready-prepared medicinal product placed on the market under a special name and in a special pack.<br>2. Medicinal product: Any substance or combination of substances presented for treating or preventing disease in human beings. Any substance or combination of substances which may be administered to human beings with a view to making a medical diagnosis or to restoring, correcting or modifying physiological functions in human beings is likewise considered a medicinal product.<br>Art 10(1). In derogation of Article 8(3)(i), and without prejudice to the law relating to the protection of industrial and commercial property:<br>(a) The applicant shall not be required to provide the results of toxicological and pharmacological tests or the results of clinical trials if he can demonstrate:<br>(i) either that the medicinal product is essentially similar to a medicinal product authorized in the Member State concerned by the application and that the holder of the marketing authorization for the original medicinal product has consented to the toxicological, pharmacological and/or clinical references contained in the file on the original medicinal product being used for the purpose of examining the application in question;<br>(ii) or that the constituent or constituents of the medicinal product have a well established medicinal use, with recognized efficacy and an acceptable level of safety, by means of a detailed scientific bibliography;<br>(iii) or that the medicinal product is essentially similar to a medicinal product which has been authorized within the Community, in accordance with Community provisions in force, for not less than six years and is marketed in the Member State for which the application is made. This period shall be extended to 10 years in the case of high-technology medicinal products having been authorised according to the procedure laid down in Article 2(5) of Council Directive 87/22/EEC(21). Furthermore, a Member State

				may also extend this period to 10 years by a single Decision covering all the medicinal products marketed on its territory where it considers this necessary in the interest of public health. Member States are at liberty not to apply the six-year period beyond the date of expiry of a patent protecting the original medicinal product.<br>However, where the medicinal product is intended for a different therapeutic use from that of the other medicinal products marketed or is to be administered by different routes or in different doses, the results of appropriate toxicological and pharmacological tests and/or of appropriate clinical trials must be provided.<br>(b) In the case of new medicinal products containing known constituents not hitherto used in combination for therapeutic purposes, the results of toxicological and pharmacological tests and of clinical trials relating to that combination must be provided, but it shall not be necessary to provide references relating to each individual constituent.
European Union	2001		Directive 2001/29/EC of the European Parliament and of the Council of 22 May 2001 on the harmonisation of certain aspects of copyright and related rights in the information society	Art 2. Member States shall provide for the exclusive right to authorise or prohibit direct or indirect, temporary or permanent reproduction by any means and in any form, in whole or in part:<br>(a) for authors, of their works;<br>Art 3(1). Member States shall provide authors with the exclusive right to authorise or prohibit any communication to the public of their works, by wire or wireless means, including the making available to the public of their works in such a way that members of the public may access them from a place and at a time individually chosen by them.<br>Art 5(2). Member States may provide for exceptions or limitations to the reproduction right provided for in Article 2 in the following cases:<br>(c) in respect of specific acts of reproduction made by publicly accessible libraries, educational establishments or museums, or by archives, which are not for direct or indirect economic or commercial advantage;<br>Art 5(3). Member States may provide for exceptions or limitations to the rights provided for in Articles 2 and 3 in the following cases:<br>(a) use for the sole purpose of illustration for teaching or scientific research, as long as the source, including the author's name, is indicated, unless this turns out to be impossible and to the extent justified by the non-commercial purpose to be achieved;<br>(b) uses, for the benefit of people with a disability, which are directly related to the disability and of a non-commercial nature, to the extent required by the specific disability;<br>(e) use for the purposes of public security or to ensure the proper performance or reporting of administrative, parliamentary or judicial proceedings;<br>(n) use by communication or making available, for the purpose of research or private

				study, to individual members of the public by dedicated terminals on the premises of establishments referred to in paragraph 2(c) of works and other subject-matter not subject to purchase or licensing terms which are contained in their collections;<br>(o) use in certain other cases of minor importance where exceptions or limitations already exist under national law, provided that they only concern analogue uses and do not affect the free circulation of goods and services within the Community, without prejudice to the other exceptions and limitations contained in this Article.<br>Art 6(1). Member States shall provide adequate legal protection against the circumvention of any effective technological measures, which the person concerned carries out in the knowledge, or with reasonable grounds to know, that he or she is pursuing that objective.<br>Art 6(3). For the purposes of this Directive, the expression 'technological measures' means any technology, device or component that, in the normal course of its operation, is designed to prevent or restrict acts, in respect of works or other subject-matter, which are not authorised by the rightholder of any copyright or any right related to copyright as provided for by law or the sui generis right provided for in Chapter III of Directive 96/9/EC. Technological measures shall be deemed 'effective' where the use of a protected work or other subject-matter is controlled by the rightholders through application of an access control or protection process, such as encryption, scrambling or other transformation of the work or other subject-matter or a copy control mechanism, which achieves the protection objective.
European Union	2004		Regulation (EC) No 726/2004 of the European Parliament and of the Council of 31 March 2004 laying down Community procedures for the authorisation and supervision of medicinal products for human and veterinary use and establishing a European Medicines Agency	Art 14(11). Without prejudice to the law on the protection of industrial and commercial property, medicinal products for human use which have been authorised in accordance with the provisions of this Regulation shall benefit from an eight-year period of data protection and a ten-year period of marketing protection, in which connection the latter period shall be extended to a maximum of 11 years if, during the first eight years of those ten years, the marketing authorisation holder obtains an authorisation for one or more new therapeutic indications which, during the scientific evaluation prior to their authorisation, are held to bring a significant clinical benefit in comparison with existing therapies.
European Union	2009		Directive 2009/24/EC of the European Parliament and of the Council of 23 April 2009 on the legal protection of computer programs	Art 1(1). In accordance with the provisions of this Directive, Member States shall protect computer programs, by copyright, as literary works within the meaning of the Berne Convention for the Protection of Literary and Artistic Works. For the purposes of this Directive, the term 'computer programs' shall include their preparatory design material.<br>Art 3. Protection shall be granted to all natural or legal persons eligible under national copyright legislation as applied to literary works.

				Art 4(1). Subject to the provisions of Articles 5 and 6, the exclusive rights of the rightholder within the meaning of Article 2 shall include the right to do or to authorise:<br>(a) the permanent or temporary reproduction of a computer program by any means and in any form, in part or in whole; in so far as loading, displaying, running, transmission or storage of the computer program necessitate such reproduction, such acts shall be subject to authorisation by the rightholder;<br>(b) the translation, adaptation, arrangement and any other alteration of a computer program and the reproduction of the results thereof, without prejudice to the rights of the person who alters the program;<br>(c) any form of distribution to the public, including the rental, of the original computer program or of copies thereof.
European Union	2017		Regulation (EU) 2017/745 of the European Parliament and of the Council of 5 April 2017 on medical devices, amending Directive 2001/83/EC, Regulation (EC) No 178/2002 and Regulation (EC) No 1223/2009 and repealing Council Directives 90/385/EEC and 93/42/EEC (Text with EEA relevance)	Art 109(1). Unless otherwise provided for in this Regulation and without prejudice to existing national provisions and practices in the Member States on confidentiality, all parties involved in the application of this Regulation shall respect the confidentiality of information and data obtained in carrying out their tasks in order to protect the following:<br>(b) commercially confidential information and trade secrets of a natural or legal person, including intellectual property rights; unless disclosure is in the public interest;
European Union	2017		Regulation (EU) 2017/746 of the European Parliament and of the Council of 5 April 2017 on in vitro diagnostic medical devices and repealing Directive 98/79/EC and Commission Decision 2010/227/EU	Art 102(1). Unless otherwise provided for in this Regulation and without prejudice to existing national provisions and practices in the Member States on confidentiality, all parties involved in the application of this Regulation shall respect the confidentiality of information and data obtained in carrying out their tasks in order to protect the following:<br>(b) commercially confidential information and trade secrets of a natural or legal person, including intellectual property rights unless disclosure is in the public interest;
European Union	2019		Directive (EU) 2019/790 of the European Parliament and of the Council of 17 April 2019 on copyright and related rights in the Digital Single Market and amending Directives 96/9/EC and 2001/29/EC	Art 2. For the purposes of this Directive, the following definitions apply:<br>(1) 'research organisation' means a university, including its libraries, a research institute or any other entity, the primary goal of which is to conduct scientific research or to carry out educational activities involving also the conduct of scientific research:<br>(a) on a not-for-profit basis or by reinvesting all the profits in its scientific research; or<br>(b) pursuant to a public interest mission recognised by a Member State;<br>in such a way that the access to the results generated by such scientific research cannot be enjoyed on a preferential basis by an undertaking that exercises a decisive

				influence upon such organisation;<br>(2) ‘text and data mining’ means any automated analytical technique aimed at analysing text and data in digital form in order to generate information which includes but is not limited to patterns, trends and correlations;<br>(6) ‘online content-sharing service provider’ means a provider of an information society service of which the main or one of the main purposes is to store and give the public access to a large amount of copyright-protected works or other protected subject matter uploaded by its users, which it organises and promotes for profit-making purposes. Providers of services, such as not-for-profit online encyclopedias, not-for-profit educational and scientific repositories, open source software-developing and-sharing platforms, providers of electronic communications services as defined in Directive (EU) 2018/1972, online marketplaces, business-to-business cloud services and cloud services that allow users to upload content for their own use, are not ‘online content-sharing service providers’ within the meaning of this Directive.<br>Art 3(1). Member States shall provide for an exception to the rights provided for in Article 5(a) and Article 7(1) of Directive 96/9/EC, Article 2 of Directive 2001/29/EC, and Article 15(1) of this Directive for reproductions and extractions made by research organisations and cultural heritage institutions in order to carry out, for the purposes of scientific research, text and data mining of works or other subject matter to which they have lawful access.<br>Art 4(1). Member States shall provide for an exception or limitation to the rights provided for in Article 5(a) and Article 7(1) of Directive 96/9/EC, Article 2 of Directive 2001/29/EC, Article 4(1)(a) and (b) of Directive 2009/24/EC and Article 15(1) of this Directive for reproductions and extractions of lawfully accessible works and other subject matter for the purposes of text and data mining.<br>Art 4(2). Reproductions and extractions made pursuant to paragraph 1 may be retained for as long as is necessary for the purposes of text and data mining.<br>Art 4(3). The exception or limitation provided for in paragraph 1 shall apply on condition that the use of works and other subject matter referred to in that paragraph has not been expressly reserved by their rightholders in an appropriate manner, such as machine-readable means in the case of content made publicly available online.
European Union	2025		Regulation (EU) 2025/327 of the European Parliament and of the Council of 11 February 2025 on the European Health Data Space and amending Directive 2011/24/EU and Regulation	Art. 33a(1). Electronic health data protected by intellectual property rights, trade secrets and/or covered by the regulatory data protection right provided by Article 10(1) of Directive 2001/83/EC or Article 14(11) of Regulation (EC) 726/2004 shall be made available for secondary use in accordance with the principles set forth in this Regulation. In this respect, the following shall apply:<br>(a) health data holders shall inform the health data access body of and identify any

			(EU) 2024/2847 (Text with EEA relevance)	electronic health data containing content or information protected by intellectual property rights, or trade secrets and/or covered by the regulatory data protection right provided by Article 10(1) of Directive 2001/83/EC or Article 14(11) of Regulation (EC) 726/2004. They shall indicate which parts of the datasets are concerned and justify why the data needs the specific protection which the data benefits from. This information shall be provided when communicating to the health data access body the dataset descriptions pursuant to Article 41(2) for the datasets it holds, or at the latest following a request received from the health data access body;<br>(b) health data access bodies shall take all specific appropriate and proportionate measures, including legal, organisational, and technical ones, they deem necessary to preserve the protection of intellectual property rights, trade secrets and/or the regulatory data protection right provided by Article 10(1) of Directive 2001/83/EC or Article 14(11) of Regulation (EC) 726/2004. the determination of the necessity and appropriateness of such measures shall rest with the health data access body;<br>(c) when issuing data permits, health data access bodies may condition the access to certain electronic health data to legal, organisational, and technical measures. Such measures may include contractual arrangements between health data holders and health data users in order to share data containing information or content protected by intellectual property rights or trade secrets. The Commission shall develop and recommend non-binding model contractual terms for such arrangements;<br>(d) should the granting of access of electronic health data for secondary purpose incur a serious risk that cannot be addressed in a satisfactory manner of infringing the intellectual property rights, trade secrets and/or the regulatory data protection right provided by Article 10(1) of Directive 2001/83/EC or Article 14(11) of Regulation (EC) 726/2004, the health data access body shall refuse access to the health data user in that respect. The health data access body shall inform the health data user of this refusal and explain why it is not possible to provide access. Health data holders and health data users shall have the right to lodge a complaint in accordance with Article 38b.
European Union	2022		Regulation (EU) 2022/868 of the European Parliament and of the Council of 30 May 2022 on European data governance and amending Regulation (EU) 2018/1724 (Data Governance Act) (1/2)	Recital 10. The categories of data held by public sector bodies which should be subject to re-use under this Regulation fall outside the scope of Directive (EU) 2019/1024 that excludes data which is not accessible due to commercial and statistical confidentiality and data that is included in works or other subject matter over which third parties have intellectual property rights. Commercially confidential data includes data protected by trade secrets, protected know-how and any other information the undue disclosure of which would have an impact on the market position or financial health of the undertaking. This Regulation should apply to personal data that fall outside the scope

				of Directive (EU) 2019/1024 insofar as the access regime excludes or restricts access to such data for reasons of data protection, privacy and the integrity of the individual, in particular in accordance with data protection rules. The re-use of data, which may contain trade secrets, should take place without prejudice to Directive (EU) 2016/943, which sets out the framework for the lawful acquisition, use or disclosure of trade secrets.<br>Recital 17. The intellectual property rights of third parties should not be affected by this Regulation. This Regulation should neither affect the existence or ownership of intellectual property rights of public sector bodies nor limit the exercise of those rights in any way. The obligations imposed in accordance with this Regulation should apply only insofar as they are compatible with international agreements on the protection of intellectual property rights, in particular the Berne Convention for the Protection of Literary and Artistic Works (Berne Convention), the Agreement on Trade-related Aspects of Intellectual Property Rights (TRIPS Agreement) and the World Intellectual Property Organization Copyright Treaty (WCT), and Union or national intellectual property law. Public sector bodies should, however, exercise their copyright in a way that facilitates re-use.<br>Recital 18. Data subject to intellectual property rights as well as trade secrets should be transmitted to a third party only where such transmission is lawful by virtue of Union or national law or with the agreement of the rights holder. Where public sector bodies are holders of the right of the maker of a database provided for in Article 7(1) of Directive 96/9/EC of the European Parliament and of the Council they should not exercise that right in order to prevent the re-use of data or to restrict re-use beyond the limits set by this Regulation.
European Union	2022		Regulation (EU) 2022/868 of the European Parliament and of the Council of 30 May 2022 on European data governance and amending Regulation (EU) 2018/1724 (Data Governance Act) (2/2)	Art 3(1). This Chapter applies to data held by public sector bodies which are protected on grounds of:<br>(a) commercial confidentiality, including business, professional and company secrets;<br>(c) the protection of intellectual property rights of third parties;<br>Art 5(2). Conditions for re-use shall be non-discriminatory, transparent, proportionate and objectively justified with regard to the categories of data and the purposes of re-use and the nature of the data for which re-use is allowed. Those conditions shall not be used to restrict competition.<br>Art 5(3). Public sector bodies shall, in accordance with Union and national law, ensure that the protected nature of data is preserved. They may provide for the following requirements:<br>(a) to grant access for the re-use of data only where the public sector body or the competent body, following the request for re-use, has ensured that data has been:

				(ii) modified, aggregated or treated by any other method of disclosure control, in the case of commercially confidential information, including trade secrets or content protected by intellectual property rights;<br>Art 5(7). Re-use of data shall be allowed only in compliance with intellectual property rights. The right of the maker of a database as provided for in Article 7(1) of Directive 96/9/EC shall not be exercised by public sector bodies in order to prevent the re-use of data or to restrict re-use beyond the limits set by this Regulation.<br>Art 7(1). For the purpose of carrying out the tasks referred to in this Article, each Member State shall designate one or more competent bodies, which may be competent for particular sectors, to assist the public sector bodies which grant or refuse access for the re-use of the categories of data referred to in Article 3(1). Member States may either establish one or more new competent bodies or rely on existing public sector bodies or on internal services of public sector bodies that fulfil the conditions laid down in this Regulation.<br>Art 7(3). The competent bodies shall have adequate legal, financial, technical and human resources to carry out the tasks assigned to them, including the necessary technical knowledge to be able to comply with relevant Union or national law concerning the access regimes for the categories of data referred to in Article 3(1).<br>Art 7(4). The assistance provided for in paragraph 1 shall include, where necessary:<br>(c) providing technical support for pseudonymisation and ensuring data processing in a manner that effectively preserves the privacy, confidentiality, integrity and accessibility of the information contained in the data for which re-use is allowed, including techniques for the anonymisation, generalisation, suppression and randomisation of personal data or other state-of-the-art privacy-preserving methods, and the deletion of commercially confidential information, including trade secrets or content protected by intellectual property rights;
European Union	2016		Directive (EU) 2016/943 of the European Parliament and of the Council of 8 June 2016 on the protection of undisclosed know-how and business information (trade secrets) against their unlawful acquisition, use and disclosure	Recital 21. Such tailoring of measures, procedures and remedies should not jeopardise or undermine fundamental rights and freedoms or the public interest, such as public safety, consumer protection, public health and environmental protection, and should be without prejudice to the mobility of workers.<br>Art 2(1). 'trade secret' means information which meets all of the following requirements:<br>(a) it is secret in the sense that it is not, as a body or in the precise configuration and assembly of its components, generally known among or readily accessible to persons within the circles that normally deal with the kind of information in question;<br>(b) it has commercial value because it is secret;<br>(c) it has been subject to reasonable steps under the circumstances, by the person

				lawfully in control of the information, to keep it secret;<br>Art 3(1). The acquisition of a trade secret shall be considered lawful when the trade secret is obtained by any of the following means:<br>(a) independent discovery or creation;<br>(b) observation, study, disassembly or testing of a product or object that has been made available to the public or that is lawfully in the possession of the acquirer of the information who is free from any legally valid duty to limit the acquisition of the trade secret;<br>(c) exercise of the right of workers or workers' representatives to information and consultation in accordance with Union law and national laws and practices;<br>(d) any other practice which, under the circumstances, is in conformity with honest commercial practices.<br>Art 3(2). The acquisition, use or disclosure of a trade secret shall be considered lawful to the extent that such acquisition, use or disclosure is required or allowed by Union or national law.<br>Art 5. Member States shall ensure that an application for the measures, procedures and remedies provided for in this Directive is dismissed where the alleged acquisition, use or disclosure of the trade secret was carried out in any of the following cases:<br>(a) for exercising the right to freedom of expression and information as set out in the Charter, including respect for the freedom and pluralism of the media;<br>(b) for revealing misconduct, wrongdoing or illegal activity, provided that the respondent acted for the purpose of protecting the general public interest;<br>(d) for the purpose of protecting a legitimate interest recognised by Union or national law.
European Union	2023		Regulation (EU) 2023/2854 of the European Parliament and of the Council of 13 December 2023 on harmonised rules on fair access to and use of data and amending Regulation (EU) 2017/2394 and Directive (EU) 2020/1828 (Data Act)	Art 1(8). This Regulation is without prejudice to Union and national legal acts providing for the protection of intellectual property rights, in particular Directives 2001/29/EC, 2004/48/EC and (EU) 2019/790.<br>Art 2(5). ‘connected product’ means an item that obtains, generates or collects data concerning its use or environment and that is able to communicate product data via an electronic communications service, physical connection or on-device access, and whose primary function is not the storing, processing or transmission of data on behalf of any party other than the user;<br>Art 2(6). ‘related service’ means a digital service, other than an electronic communications service, including software, which is connected with the product at the time of the purchase, rent or lease in such a way that its absence would prevent the connected product from performing one or more of its functions, or which is subsequently connected to the product by the manufacturer or a third party to add to,

				update or adapt the functions of the connected product;<br>Art 2(38). 'exportable data', for the purpose of Articles 23 to 31 and Article 35, means the input and output data, including metadata, directly or indirectly generated, or cogenerated, by the customer's use of the data processing service, excluding any assets or data protected by intellectual property rights, or constituting a trade secret, of providers of data processing services or third parties;<br>Art 4(6). Trade secrets shall be preserved and shall be disclosed only where the data holder and the user take all necessary measures prior to the disclosure to preserve their confidentiality in particular regarding third parties. The data holder or, where they are not the same person, the trade secret holder shall identify the data which are protected as trade secrets, including in the relevant metadata, and shall agree with the user proportionate technical and organisational measures necessary to preserve the confidentiality of the shared data, in particular in relation to third parties, such as model contractual terms, confidentiality agreements, strict access protocols, technical standards and the application of codes of conduct.<br>Art 4(7). Where there is no agreement on the necessary measures referred to in paragraph 6, or if the user fails to implement the measures agreed pursuant to paragraph 6 or undermines the confidentiality of the trade secrets, the data holder may withhold or, as the case may be, suspend the sharing of data identified as trade secrets. The decision of the data holder shall be duly substantiated and provided in writing to the user without undue delay. In such cases, the data holder shall notify the competent authority designated pursuant to Article 37 that it has withheld or suspended data sharing and identify which measures have not been agreed or implemented and, where relevant, which trade secrets have had their confidentiality undermined.<br>Art 4(8). In exceptional circumstances, where the data holder who is a trade secret holder is able to demonstrate that it is highly likely to suffer serious economic damage from the disclosure of trade secrets, despite the technical and organisational measures taken by the user pursuant to paragraph 6 of this Article, that data holder may refuse on a case-by-case basis a request for access to the specific data in question. That demonstration shall be duly substantiated on the basis of objective elements, in particular the enforceability of trade secrets protection in third countries, the nature and level of confidentiality of the data requested, and the uniqueness and novelty of the connected product, and shall be provided in writing to the user without undue delay. Where the data holder refuses to share data pursuant to this paragraph, it shall notify the competent authority designated pursuant to Article 37.<br>Art 13(3). A contractual term is unfair if it is of such a nature that its use grossly

				deviates from good commercial practice in data access and use, contrary to good faith and fair dealing.<br>Art 13(5). A contractual term shall be presumed to be unfair for the purposes of paragraph 3 if its object or effect is to:<br>(b) allow the party that unilaterally imposed the term to access and use the data of the other contracting party in a manner that is significantly detrimental to the legitimate interests of the other contracting party, in particular when such data contain commercially sensitive data or are protected by trade secrets or by intellectual property rights;<br>Art 30(6). Providers of data processing services shall not be required to develop new technologies or services, or disclose or transfer digital assets that are protected by intellectual property rights or that constitute a trade secret, to a customer or to a different provider of data processing services or compromise the customer's or provider's security and integrity of service.<br>Art 43. The sui generis right provided for in Article 7 of Directive 96/9/EC shall not apply when data is obtained from or generated by a connected product or related service falling within the scope of this Regulation, in particular in relation to Articles 4 and 5 thereof.
European Union	2014		Regulation (EU) No 536/2014 of the European Parliament and of the Council of 16 April 2014 on clinical trials on medicinal products for human use, and repealing Directive 2001/20/EC (Clinical Trials Regulation)	Art 81(1). The Agency shall, in collaboration with the Member States and the Commission, set up and maintain a EU database at Union level. The Agency shall be considered to be the controller of the EU database and shall be responsible for avoiding unnecessary duplication between the EU database and the EudraCT and Eudravigilance databases. The EU database shall contain the data and information submitted in accordance with this Regulation. The EU database shall identify each clinical trial by a unique EU trial number. The sponsor shall refer to this EU trial number in any subsequent submission relating or referring to that clinical trial.<br>Art 81(4). The EU database shall be publicly accessible unless, for all or part of the data and information contained therein, confidentiality is justified on any of the following grounds:<br>(a) protecting personal data in accordance with Regulation (EC) No 45/2001;<br>(b) protecting commercially confidential information, in particular through taking into account the status of the marketing authorisation for the medicinal product, unless there is an overriding public interest in disclosure;
European Union	2024		Regulation (EU) 2024/1689 of the European Parliament and of the Council of 13 June 2024 laying down harmonised rules	Art 11(1). The technical documentation of a high-risk AI system shall be drawn up before that system is placed on the market or put into service and shall be kept up-to date. The technical documentation shall be drawn up in such a way as to demonstrate that the high-risk AI system complies with the requirements set out in this Section and

			on artificial intelligence and amending Regulations (EC) No 300/2008, (EU) No 167/2013, (EU) No 168/2013, (EU) 2018/858, (EU) 2018/1139 and (EU) 2019/2144 and Directives 2014/90/EU, (EU) 2016/797 and (EU) 2020/1828 (Artificial Intelligence Act) (1/3)	to provide national competent authorities and notified bodies with the necessary information in a clear and comprehensive form to assess the compliance of the AI system with those requirements. It shall contain, at a minimum, the elements set out in Annex IV. SMEs, including start-ups, may provide the elements of the technical documentation specified in Annex IV in a simplified manner. To that end, the Commission shall establish a simplified technical documentation form targeted at the needs of small and microenterprises. Where an SME, including a start-up, opts to provide the information required in Annex IV in a simplified manner, it shall use the form referred to in this paragraph. Notified bodies shall accept the form for the purposes of the conformity assessment.<br>Art 11(2). Where a high-risk AI system related to a product covered by the Union harmonisation legislation listed in Section A of Annex I is placed on the market or put into service, a single set of technical documentation shall be drawn up containing all the information set out in paragraph 1, as well as the information required under those legal acts.<br>Art 13(1). High-risk AI systems shall be designed and developed in such a way as to ensure that their operation is sufficiently transparent to enable deployers to interpret a system's output and use it appropriately. An appropriate type and degree of transparency shall be ensured with a view to achieving compliance with the relevant obligations of the provider and deployer set out in Section 3.<br>Art 13(2). High-risk AI systems shall be accompanied by instructions for use in an appropriate digital format or otherwise that include concise, complete, correct and clear information that is relevant, accessible and comprehensible to deployers.
European Union	2024		Regulation (EU) 2024/1689 of the European Parliament and of the Council of 13 June 2024 laying down harmonised rules on artificial intelligence and amending Regulations (EC) No 300/2008, (EU) No 167/2013, (EU) No 168/2013, (EU) 2018/858, (EU) 2018/1139 and (EU) 2019/2144 and Directives 2014/90/EU, (EU) 2016/797 and (EU) 2020/1828 (Artificial Intelligence Act) (2/3)	Art 53(1). Providers of general-purpose AI models shall:<br>(b) draw up, keep up-to-date and make available information and documentation to providers of AI systems who intend to integrate the general-purpose AI model into their AI systems. Without prejudice to the need to observe and protect intellectual property rights and confidential business information or trade secrets in accordance with Union and national law, the information and documentation shall:<br>(i) enable providers of AI systems to have a good understanding of the capabilities and limitations of the general-purpose AI model and to comply with their obligations pursuant to this Regulation; and<br>(ii) contain, at a minimum, the elements set out in Annex XII;<br>(c) put in place a policy to comply with Union law on copyright and related rights, and in particular to identify and comply with, including through state-of-the-art technologies, a reservation of rights expressed pursuant to Article 4(3) of Directive (EU) 2019/790;

				Art 53(7). Any information or documentation obtained pursuant to this Article, including trade secrets, shall be treated in accordance with the confidentiality obligations set out in Article 78.<br>Art 78(1). The Commission, market surveillance authorities and notified bodies and any other natural or legal person involved in the application of this Regulation shall, in accordance with Union or national law, respect the confidentiality of information and data obtained in carrying out their tasks and activities in such a manner as to protect, in particular:<br>(a) the intellectual property rights and confidential business information or trade secrets of a natural or legal person, including source code, except in the cases referred to in Article 5 of Directive (EU) 2016/943 of the European Parliament and of the Council;<br>(b) the effective implementation of this Regulation, in particular for the purposes of inspections, investigations or audits;<br>(c) public and national security interests;<br>(d) the conduct of criminal or administrative proceedings;<br>(e) information classified pursuant to Union or national law.
European Union	2024		Regulation (EU) 2024/1689 of the European Parliament and of the Council of 13 June 2024 laying down harmonised rules on artificial intelligence and amending Regulations (EC) No 300/2008, (EU) No 167/2013, (EU) No 168/2013, (EU) 2018/858, (EU) 2018/1139 and (EU) 2019/2144 and Directives 2014/90/EU, (EU) 2016/797 and (EU) 2020/1828 (Artificial Intelligence Act) (3/3)	Annex IV. The technical documentation referred to in Article 11(1) shall contain at least the following information, as applicable to the relevant AI system:<br>1. A general description of the AI system including:<br>(a) its intended purpose, the name of the provider and the version of the system reflecting its relation to previous versions;<br>(b) how the AI system interacts with, or can be used to interact with, hardware or software, including with other AI systems, that are not part of the AI system itself, where applicable;<br>(c) the versions of relevant software or firmware, and any requirements related to version updates;<br>(d) the description of all the forms in which the AI system is placed on the market or put into service, such as software packages embedded into hardware, downloads, or APIs;<br>(e) the description of the hardware on which the AI system is intended to run;<br>(f) where the AI system is a component of products, photographs or illustrations showing external features, the marking and internal layout of those products;<br>(g) a basic description of the user-interface provided to the deployer;<br>(h) instructions for use for the deployer, and a basic description of the user-interface provided to the deployer, where applicable;<br>2. A detailed description of the elements of the AI system and of the process for its

				development, including:<br>(a) the methods and steps performed for the development of the AI system, including, where relevant, recourse to pre-trained systems or tools provided by third parties and how those were used, integrated or modified by the provider;<br>(b) the design specifications of the system, namely the general logic of the AI system and of the algorithms; the key design choices including the rationale and assumptions made, including with regard to persons or groups of persons in respect of who, the system is intended to be used; the main classification choices; what the system is designed to optimise for, and the relevance of the different parameters; the description of the expected output and output quality of the system; the decisions about any possible trade-off made regarding the technical solutions adopted to comply with the requirements set out in Chapter III, Section 2;<br>(c) the description of the system architecture explaining how software components build on or feed into each other and integrate into the overall processing; the computational resources used to develop, train, test and validate the AI system;<br>(d) where relevant, the data requirements in terms of datasheets describing the training methodologies and techniques and the training data sets used, including a general description of these data sets, information about their provenance, scope and main characteristics; how the data was obtained and selected; labelling procedures (e.g. for supervised learning), data cleaning methodologies (e.g. outliers detection);<br>(e) assessment of the human oversight measures needed in accordance with Article 14, including an assessment of the technical measures needed to facilitate the interpretation of the outputs of AI systems by the deployers, in accordance with Article 13(3), point (d);<br>(f) where applicable, a detailed description of pre-determined changes to the AI system and its performance, together with all the relevant information related to the technical solutions adopted to ensure continuous compliance of the AI system with the relevant requirements set out in Chapter III, Section 2;<br>(g) the validation and testing procedures used, including information about the validation and testing data used and their main characteristics; metrics used to measure accuracy, robustness and compliance with other relevant requirements set out in Chapter III, Section 2, as well as potentially discriminatory impacts; test logs and all test reports dated and signed by the responsible persons, including with regard to pre-determined changes as referred to under point (f);<br>(h) cybersecurity measures put in place;<br>Annex XII. The information referred to in Article 53(1), point (b) shall contain at least the following:

				1. A general description of the general-purpose AI model including:<br>(a) the tasks that the model is intended to perform and the type and nature of AI systems into which it can be integrated;<br>(b) the acceptable use policies applicable;<br>(c) the date of release and methods of distribution;<br>(d) how the model interacts, or can be used to interact, with hardware or software that is not part of the model itself, where applicable;<br>(e) the versions of relevant software related to the use of the general-purpose AI model, where applicable;<br>(f) the architecture and number of parameters;<br>(g) the modality (e.g. text, image) and format of inputs and outputs;<br>(h) the licence for the model.<br>2. A description of the elements of the model and of the process for its development, including:<br>(a) the technical means (e.g. instructions for use, infrastructure, tools) required for the general-purpose AI model to be integrated into AI systems;<br>(b) the modality (e.g. text, image, etc.) and format of the inputs and outputs and their maximum size (e.g. context window length, etc.);<br>(c) information on the data used for training, testing and validation, where applicable, including the type and provenance of data and curation methodologies.
European Union	2016		Regulation (EU) 2016/679 of the European Parliament and of the Council of 27 April 2016 on the protection of natural persons with regard to the processing of personal data and on the free movement of such data, and repealing Directive 95/46/EC (General Data Protection Regulation)	Recital 63. A data subject should have the right of access to personal data which have been collected concerning him or her, and to exercise that right easily and at reasonable intervals, in order to be aware of, and verify, the lawfulness of the processing. This includes the right for data subjects to have access to data concerning their health, for example the data in their medical records containing information such as diagnoses, examination results, assessments by treating physicians and any treatment or interventions provided. Every data subject should therefore have the right to know and obtain communication in particular with regard to the purposes for which the personal data are processed, where possible the period for which the personal data are processed, the recipients of the personal data, the logic involved in any automatic personal data processing and, at least when based on profiling, the consequences of such processing. Where possible, the controller should be able to provide remote access to a secure system which would provide the data subject with direct access to his or her personal data. That right should not adversely affect the rights or freedoms of others, including trade secrets or intellectual property and in particular the copyright protecting the software. However, the result of those considerations should not be a refusal to provide all information to the data subject. Where the controller processes a

				large quantity of information concerning the data subject, the controller should be able to request that, before the information is delivered, the data subject specify the information or processing activities to which the request relates.<br>Art 4(15). Data concerning health' means personal data related to the physical or mental health of a natural person, including the provision of health care services, which reveal information about his or her health status<br>Art 9(1). Processing of personal data revealing racial or ethnic origin, political opinions, religious or philosophical beliefs, or trade union membership, and the processing of genetic data, biometric data for the purpose of uniquely identifying a natural person, data concerning health or data concerning a natural person's sex life or sexual orientation shall be prohibited.<br>Art 9(2). Paragraph 1 shall not apply if one of the following applies:<br>(a) the data subject has given explicit consent to the processing of those personal data for one or more specified purposes, except where Union or Member State law provide that the prohibition referred to in paragraph 1 may not be lifted by the data subject;<br>(b) processing is necessary for the purposes of carrying out the obligations and exercising specific rights of the controller or of the data subject in the field of employment and social security and social protection law in so far as it is authorised by Union or Member State law or a collective agreement pursuant to Member State law providing for appropriate safeguards for the fundamental rights and the interests of the data subject;<br>(c) processing is necessary to protect the vital interests of the data subject or of another natural person where the data subject is physically or legally incapable of giving consent;<br>(d) processing is carried out in the course of its legitimate activities with appropriate safeguards by a foundation, association or any other not-for-profit body with a political, philosophical, religious or trade union aim and on condition that the processing relates solely to the members or to former members of the body or to persons who have regular contact with it in connection with its purposes and that the personal data are not disclosed outside that body without the consent of the data subjects;<br>(e) processing relates to personal data which are manifestly made public by the data subject;<br>(f) processing is necessary for the establishment, exercise or defence of legal claims or whenever courts are acting in their judicial capacity;<br>(g) processing is necessary for reasons of substantial public interest, on the basis of Union or Member State law which shall be proportionate to the aim pursued, respect

				the essence of the right to data protection and provide for suitable and specific measures to safeguard the fundamental rights and the interests of the data subject;<br>(h) processing is necessary for the purposes of preventive or occupational medicine, for the assessment of the working capacity of the employee, medical diagnosis, the provision of health or social care or treatment or the management of health or social care systems and services on the basis of Union or Member State law or pursuant to contract with a health professional and subject to the conditions and safeguards referred to in paragraph 3;<br>(i) processing is necessary for reasons of public interest in the area of public health, such as protecting against serious cross-border threats to health or ensuring high standards of quality and safety of health care and of medicinal products or medical devices, on the basis of Union or Member State law which provides for suitable and specific measures to safeguard the rights and freedoms of the data subject, in particular professional secrecy;<br>(j) processing is necessary for archiving purposes in the public interest, scientific or historical research purposes or statistical purposes in accordance with Article 89(1) based on Union or Member State law which shall be proportionate to the aim pursued, respect the essence of the right to data protection and provide for suitable and specific measures to safeguard the fundamental rights and the interests of the data subject.<br>Art 9(3). Personal data referred to in paragraph 1 may be processed for the purposes referred to in point (h) of paragraph 2 when those data are processed by or under the responsibility of a professional subject to the obligation of professional secrecy under Union or Member State law or rules established by national competent bodies or by another person also subject to an obligation of secrecy under Union or Member State law or rules established by national competent bodies.<br>Art 22(1). The data subject shall have the right not to be subject to a decision based solely on automated processing, including profiling, which produces legal effects concerning him or her or similarly significantly affects him or her.<br>Art 35(9). Where appropriate, the controller shall seek the views of data subjects or their representatives on the intended processing, without prejudice to the protection of commercial or public interests or the security of processing operations.
France	1992	2024	Intellectual Property Code	Art L111-1. The author of a work of the mind enjoys on this work, solely because of its creation, an exclusive and enforceable intangible property right against all. This right includes intellectual and moral attributes as well as patrimonial attributes, which are determined by Books I and III of this Code.<br>Art L112-1. The provisions of this Code protect the rights of authors to all works of the

				mind, regardless of genre, form of expression, merit or destination.<br>Art L112-2. The following are considered in particular as works of the mind within the meaning of this Code:<br>1° Books, brochures and other literary, artistic and scientific writings;<br>13° Software, including preparatory design hardware;<br>Art L112-3. The authors of translations, adaptations, transformations or arrangements of works of the mind shall enjoy the protection established by this Code without prejudice to the rights of the author of the original work. The same applies to authors of anthologies or collections of works or various data, such as databases, which, by the choice or arrangement of materials, constitute intellectual creations. A database means a collection of works, data or other independent elements, arranged in a systematic or methodical manner, and individually accessible by electronic means or by any other means.<br>Art L113-1. The status of author belongs, unless proven otherwise, to the person or those under the name of whom the work is disclosed.<br>Art L122-5. When the work has been disclosed, the author cannot prohibit:<br>3° Provided that the name of the author and the source are clearly indicated:<br>e) The representation or reproduction of extracts from works, subject to works designed for educational purposes and musical scores, for exclusive illustration purposes in the context of research, provided that this representation or reproduction is intended, in particular by means of a digital workspace, for a majority audience composed of researchers directly concerned by the research activity requiring this representation or reproduction, that it is not published or disseminated to a third party to the public thus constituted, that the use of this representation or reproduction does not give rise to any commercial exploitation and that it is compensated by remuneration negotiated on a flat-rate basis without prejudice to the transfer of the right of reproduction by reprography mentioned in Article L. 122-10;<br>6° The provisional reproduction of a transitional or incidental nature, when it is an integral and essential part of a technical process and its sole purpose is to allow the lawful use of the work or its transmission between third parties through a network using an intermediary; however, this provisional reproduction, which may only relate to works other than software and databases, must not have its own economic value;<br>10° Digital copies or reproductions of a work for text and data mining carried out under the conditions provided for in Article L. 122-5-3;<br>Art L122-5-3.<br>I.-Text and data mining, within the meaning of 10° of Article L. 122-5, means the implementation of an automated analysis technique of text and data in digital form in

				order to obtain information, in particular constants, trends and correlations.<br>II.-Digital copies or reproductions of works that have been accessed lawfully may be made without the authorization of authors for excavations of texts and data carried out solely for the purpose of scientific research by research organizations, libraries accessible to the public, museums, archive services or institutions holding cinematographic, audiovisual or sound heritage, or on their behalf and at their request by other persons, including in the framework of a non-profit partnership with private actors.<br><br>The provisions of the previous paragraph shall not apply when a company, shareholder or partner of the body or institution carrying out the excavations has privileged access to their results.<br><br>Digital copies and reproductions made during a text and data excavation are stored with an appropriate level of security and may be kept for the exclusive purpose of scientific research, including for the verification of research results.<br><br>Copyright holders may implement proportionate and necessary measures to ensure the security and integrity of the networks and databases in which the works are hosted.<br><br>An agreement concluded between the organizations representing copyright holders and the bodies and institutions mentioned in the first paragraph of this II may define good practice relating to the implementation of its provisions.<br><br>III.-Without prejudice to the provisions of II, digital copies or reproductions of works to which it has been accessed lawfully may be made for the purpose of excavations of texts and data carried out by any person, regardless of the purpose of the search, unless the author has objected appropriately, in particular by machine-readable processes for content made available to the public online. Copies and reproductions are stored with an appropriate level of security and then destroyed after the text and data excavation.<br>Art L122-6. Subject to the provisions of Article L. 122-6-1, the right of exploitation belonging to the author of a software includes the right to perform and authorize:<br>1° The permanent or temporary reproduction of a software in whole or in part by any means and in any form. To the extent that the loading, display, execution, transmission or storage of this software requires reproduction, these acts are only possible with the

				authorization of the author;<br>2° The translation, adaptation, arrangement or any other modification of a software and the reproduction of the resulting software;<br>3° The placing on the market for a fee or free of charge, including the rental, of the copy or copies of a software by any process. However, the first sale of a copy of a software in the territory of a Member State of the European Community or a State party to the Agreement on the European Economic Area by the author or with his consent exhausts the right to put that copy on the market in all Member States with the exception of the right to authorize the subsequent rental of a copy.<br>Art L123-1. The author enjoys, throughout his life, the exclusive right to exploit his work in any form whatsoever and to make a financial profit from it. Upon the death of the author, this right persists for the benefit of his beneficiaries during the current calendar year and the following seventy years.
				Art L341-1. The producer of a database, understood as the person who takes the initiative and the risk of the corresponding investments, benefits from protection of the content of the database when the constitution, verification or presentation of it attests to a substantial financial, material or human investment. This protection is independent and is without prejudice to those resulting from copyright or other right in the database or one of its constituent elements.<br>Art L341-2. The following are eligible for the benefit of this title:<br>1° Producers of databases, nationals of a Member State of the European Community or of a State party to the Agreement on the European Economic Area, or who have their habitual residence in such a State;<br>2° Companies or enterprises incorporated in accordance with the legislation of a Member State and having their registered office, central administration or principal place of business within the Community or a State party to the Agreement on the European Economic Area; nevertheless, if such a company or undertaking has only its registered office in the territory of such a State, its activities must have a real and continuous link with the economy of one of them.<br><br>Producers of databases who do not meet the conditions mentioned above are admitted to the protection provided for in this Title when a special agreement has been concluded with the State of which they are nationals by the Council of the European Community.<br>Art L342-1. The database producer has the right to prohibit:<br>1° The extraction, by permanent or temporary transfer of all or a qualitatively or quantitatively substantial part of the content of a database to another medium, by any

				means and in any form whatsoever;<br>2° The reuse, by making available to the public of all or a qualitatively or quantitatively substantial part of the content of the database, whatever its form.<br><br>These rights can be transferred or assigned or licensed. The public loan is not an act of extraction or reuse.<br>Art L342-2. The producer may also prohibit the repeated and systematic extraction or reuse of qualitatively or quantitatively non-substantial parts of the content of the database when these operations clearly exceed the conditions of normal use of the database.<br>Art L342-3. When a database is made available to the public by the rights holder, the right holder cannot prohibit:<br>1° The extraction or reuse of a non-substantial part, assessed qualitatively or quantitatively, of the content of the database, by the person who has legal access to it;<br>2° The extraction for private purposes of a qualitatively or quantitatively substantial part of the content of a non-electronic database subject to respect for copyright or related rights on the works or elements incorporated in the database;<br>3° The extraction and reuse of a database under the conditions defined in 7° of Article L. 122-5, 1° of Article L. 122-5-1 and Article L. 122-5-2;<br>4° The extraction and reuse of a substantial part, appreciated qualitatively or quantitatively, of the content of the database, subject to databases designed for educational purposes and databases created for a digital edition of the written, for the exclusive purposes of illustration in the context of research, to the exclusion of any playful or recreational activity, provided that the public for which this extraction and reuse are intended is composed mainly of researchers directly concerned, that the source is indicated, that the use of this extraction and reuse does not give rise to any exploitation commercial and that it is compensated by remuneration negotiated on a flat-rate basis;<br>4° bis The extraction and reuse of a substantial part, assessed qualitatively or quantitatively, of the content of the database for exclusive illustration purposes in the context of vocational education and training, under the conditions provided for in Article L. 122-5-4. For the application of this article, the author means the beneficiary of the rights and the representation and reproduction of extracts from works means the extraction and reuse of a substantial part of a database;<br>5° The extraction and reuse of a database under the conditions defined in 8° of Article L. 122-5;<br>6° Digital extractions, copies or reproductions of a database, for the search of texts

				and data carried out under the conditions provided for in Article L. 122-5-3. For the application of this article, authors and copyright holders mean database producers and digital copies or reproductions of works mean digital extractions, copies or reproductions of databases;<br>7° The extraction and reuse of a database under the conditions defined in 13° of Article L. 122-5.<br><br>Any clause contrary to 1° or 6° above is null and void. The exceptions listed in this article may not prejudice the normal operation of the database or cause unjustified harm to the legitimate interests of the producer of the database. The procedures for the application of this article are specified by decree in the Council of State.
				Art L611-10.<br>1. New inventions involving an inventive activity and susceptible to industrial application are patentable in all technological fields.<br>2. The following shall not be considered as inventions within the meaning of the first paragraph of this Article in particular:<br>a) Discoveries as well as scientific theories and mathematical methods;<br>b) Aesthetic creations;<br>c) Plans, principles and methods in the exercise of intellectual activities, in terms of gambling or in the field of economic activities, as well as computer programs;<br>d) Presentations of information.
				Art L611-16. Methods of surgical or therapeutic treatment of the human or animal body and the diagnostic methods applied to the human or animal body are not patentable. This provision does not apply to products, in particular substances or compositions, for the implementation of one of these methods.
Italy	1941	2023	LEGGE 22 aprile 1941, n. 633 Protezione del diritto d'autore" .Y "di altri diritti connessi al suo esercizio.	Art 1. Works of creative ingenuity belonging to literature, music, figurative arts, architecture, theater and cinematography, whatever their form or form of expression, are protected under this law. Computer programs as literary works within the meaning of the Berne Convention on the Protection of Literary and Artistic Works ratified and enforced by Law No 399 of 20 June 1978 ((, as well as databases which for the choice or arrangement of the material constitute an intellectual creation of the author) are also protected.<br>Art 2. In particular, the protection includes:<br>8) computer programs, in any form expressed as long as they are original as a result of the author's intellectual creation. The ideas and principles underlying any element of a programme, including those underlying its interfaces, shall be excluded from the protection granted by this Act. The term programme also includes preparatory material

				for the design of the programme itself.<br>9) The databases referred to in the second paragraph of Article 1, understood as collections of works, data or other independent elements systematically or methodically arranged and individually accessible by electronic means or otherwise. The protection of databases does not extend to their content and is without prejudice to existing rights to that content.<br>Art 12. The author has the exclusive right to publish the work. It also has the exclusive right to use the work economically in any form and manner, original or derivative, within the limits established by this law, and in particular by exercising the exclusive rights indicated in the following articles. The first form of exercise of the right of use shall be deemed to be the first publication.<br>Art 13. The exclusive right to reproduce shall have as its object the multiplication in direct or indirect copies, temporary or permanent, in whole or in part of the work, in any way or form, such as hand copying, printing, lithography, engraving, photography, phonography, cinematography and any other reproduction process.<br>Art 25. The rights of economic use of the work last throughout the life of the author and until 70 years after his death.<br>Art 64-bis. Subject to the provisions of Articles 64-b and 64-c, the exclusive rights conferred by this Computer Program Act shall include the right to perform or authorise:<br>a) the reproduction, permanent or temporary, in whole or in part, of the computer program by any means or in any form.<br>To the extent that operations such as uploading, displaying, executing, transmitting or storing the computer program require reproduction, such operations shall also be subject to the authorisation of the rightholder;<br>(b) the translation, adaptation, transformation and any other modification of the computer program, as well as the reproduction of the resulting work, without prejudice to the rights of the person modifying the programme;<br>(c) any form of distribution to the public, including leasing, of the original computer program or copies of it. The first sale of a copy of the programme in the European Economic Community by or with the rightholder's consent, exhausts the right to distribute that copy within the Community, with the exception of the right to control the further rental of the program or a copy of the program.<br>Art 64-quinquies. The author of a database has the exclusive right to perform or authorize:<br>(a) permanent or temporary reproduction, in whole or in part, by any means and in any form;<br>(b) the translation, the adaptation, a different provision and any other modification;

				(c) any form of distribution to the public of the original or copies of the database; the first sale of a copy in the territory of the European Union by or with his consent of the rightholder depletes the right to control, within the Union, subsequent sales of the copy;<br>(d) any presentation, demonstration or communication in public, including transmission by any means and in any form;<br>(e) any public reproduction, distribution, communication, presentation or demonstration of the results of the operations referred to in point (b).<br>Art 64-sexies. The following shall not be subject to the authorisation referred to in Article 64-quinquies by the rightholder:<br>(a) access to or consultation of the database when they are exclusively for educational or scientific research purposes, not carried out within an undertaking, provided that the source is indicated and within the limits of what is justified by the non-commercial purpose pursued.<br>In the context of such access and consultation activities, any operations of permanent reproduction of all or a substantial part of the content on another medium shall in any event be subject to the authorisation of the rightholder;
Spain	1996		Royal Legislative Decree 1/1996, of April 12, approving the consolidated text of the Intellectual Property Law, regularizing, clarifying and harmonizing the current legal provisions on the subject.	Art 1. The intellectual property of a literary, artistic or scientific work corresponds to the author for the sole fact of its creation.<br>Art 2. Intellectual property is made up of personal and patrimonial rights, which attribute to the author the full disposition and the exclusive right to the exploitation of the work, with no limitations other than those established in the Law<br>Art 5.<br>1. The natural person who creates a literary, artistic or scientific work is considered an author.<br>2. However, legal persons may benefit from the protection that this Law grants to the author in the cases expressly provided for in it.<br>Art 10.<br>1. All original literary, artistic or scientific creations expressed by any means or medium, tangible or intangible, currently known or invented in the future, are subject to intellectual property, including:<br>i) Computer programs.<br>Art 11. Without prejudice to the copyright on the original work, the following are also subject to intellectual property:<br>1.º Translations and adaptations.<br>2.º Revisions, updates and annotations.<br>3.º The compendiums, summaries and extracts.

				5.º Any transformations of a literary, artistic or scientific work.<br>Art 12.<br>1. [Collections and databases] are also object of intellectual property, under the terms of Book I of this Law, the collections of other people's works, data or other independent elements such as anthologies and databases that by the selection or disposition of their contents constitute intellectual creations, without prejudice, where appropriate, to the rights that may subsist over said contents. The protection recognized in this article to these collections refers only to their structure as a form of expression of the selection or disposition of their contents, not being extended to these.<br>2. For the purposes of this Law, and without prejudice to the provisions of the previous section, the collections of works, data, or other independent elements arranged in a systematic or methodical way and accessible individually by electronic means or otherwise are considered databases.<br>3. The protection granted to databases under this article will not apply to computer programs used in the manufacture or operation of databases accessible by electronic means.<br>Art 14. The author has the following inalienable and inalienable rights:<br>1.º Decide if your work is to be disclosed and in what form.<br>2.º Determine if such disclosure should be made with your name, under a pseudonym or sign, or anonymously.<br>3.º Require the recognition of your status as author of the work.<br>4.º Demand respect for the integrity of the work and prevent any deformation, modification, alteration or attack on it that harms its legitimate interests or impairs its reputation.<br>5.º Modify the work respecting the rights acquired by third parties and the requirements of protection of goods of cultural interest.<br>6.º Withdraw the work from commerce, by change of its intellectual or moral convictions, after compensation for damages to the holders of exploitation rights. If, subsequently, the author decides to resume the exploitation of his work, he must preferably offer the corresponding rights to the previous owner of them and under conditions reasonably similar to those original.<br>7.º Access the single or rare copy of the work, when it is in the possession of another, in order to exercise the right of disclosure or any other that corresponds to it. This right will not allow the displacement of the work to be required and access to it will be carried out in the place and form that cause less discomfort to the owner, who will be compensated, where appropriate, for the damages that are infested.

				Art 17. The author is responsible for the exclusive exercise of the rights of exploitation of his work in any form and, in particular, the rights of reproduction, distribution, public communication and transformation, which may not be carried out without his authorization, except in the cases provided for in this Law.<br>Art 18. Reproduction is understood as the direct or indirect, provisional or permanent fixation, by any means and in any form, of the whole or part of the work, that allows its communication or the obtaining of copies.<br>Art 19.<br>1. Distribution is understood as the making available to the public of the original or copies of the work, on a tangible medium, through its sale, rental, loan or in any other way.<br>2. When the distribution is made by sale or other title of transfer of the property, within the scope of the European Union, by the owner of the right himself or with his consent, this right will be exhausted with the first, although only for successive sales and transfers of property that are made in that territorial scope.<br>3. Rental is understood as the provision of the originals and copies of a work for use for a limited time and with a direct or indirect economic or commercial benefit. The provision for exhibition purposes, public communication from phonograms or audiovisual recordings, including fragments of one and the other, and the one that is made for consultation on site, are excluded from the concept of rental.<br>4. Loan is understood as the provision of originals and copies of a work for use for a limited time without direct or indirect economic or commercial benefit, provided that such loan is carried out through establishments accessible to the public. It will be understood that there is no direct or indirect economic or commercial benefit when the loan made by an establishment accessible to the public results in the payment of an amount that does not exceed what is necessary to cover the operating expenses. This amount may not include in whole or in part the amount of the remuneration right that must be paid to the holders of intellectual property rights in accordance with the provisions of article 37.2. The operations mentioned in the second paragraph of paragraph 3 and those carried out between establishments accessible to the public are excluded from the concept of loan.<br>5. The provisions of this article regarding rent and loan will not apply to buildings or applied works of arts.<br>Art 20.<br>1. Public communication will be understood as any act by which a plurality of people can have access to the work without prior distribution of copies to each of them. Communication will not be considered public when it is held within a strictly domestic

				environment that is not integrated or connected to a broadcasting network of any kind.<br>2. Especially, they are acts of public communication:<br>e) The transmission of any works to the public by wire, cable, optical fiber or other analogous procedure, whether or not by fertilizer.<br>i) The making available to the public of works, by wired or wireless procedures, in such a way that anyone can access them from the place and at the time of their choice.<br>j) Public access in any form to the works incorporated into a database, although said database is not protected by the provisions of Book I of this Law.<br>k) The performance of any of the above acts, with respect to a database protected by Book I of this Law.<br>Art 21.<br>1. The transformation of a work includes its translation, adaptation and any other modification in its form from which a different work is derived. In the case of a database referred to in article 12 of this Law, the reorganization of the same will also be considered transformation.<br>2. The intellectual property rights of the work resulting from the transformation will correspond to the author of the latter, without prejudice to the right of the author of the pre-existing work to authorize, throughout the period of protection of his rights over it, the exploitation of those results in any form and especially through its reproduction, distribution, public communication or new transformation.<br>Art 26. The rights of exploitation of the work will last the entire life of the author and seventy years after his death or declaration of death.
				Art 96.<br>1. For the purposes of this Law, a computer program shall be understood as any sequence of instructions or indications intended to be used, directly or indirectly, in a computer system to perform a function or a task or to obtain a certain result, whatever its form of expression and fixation. For the same purposes, the term computer programs will also include your preparatory documentation. The technical documentation and user manuals of a program will enjoy the same protection that this Title provides to computer programs.<br>2. The computer program will be protected only if it is original, in the sense of being an intellectual creation of its author.<br>3. The protection provided for in this Law will apply to any form of expression of a computer program. Likewise, this protection extends to any successive versions of the program as well as to derived programs, except those created in order to cause harmful effects to a computer system. When computer programs are part of a patent or a utility model, they will enjoy, without prejudice to the provisions of this Law, the

				protection that may correspond to them by application of the legal regime of industrial property.<br>4. The ideas and principles on which any of the elements of a computer program are based, including those that serve as the basis for its interfaces, will not be protected by copyright in accordance with this Law.<br>Art 97.<br>1. The author of the computer program will be the person or group of natural persons who have created it, or the legal person that is contemplated as the owner of the copyright in the cases expressly provided for by this Law.<br>2. In the case of a collective work, the natural or legal person who edits and discloses it under its name will be considered an author, unless otherwise agreed.<br>3. The copyright on a computer program that is a unitary result of the collaboration between several authors will be the common property and will correspond to all of them in the proportion they determine.<br>4. When an employee creates a computer program, in the exercise of the functions entrusted to him or following the instructions of his employer, the ownership of the exploitation rights corresponding to the computer program thus created, both the source program and the object program, will correspond exclusively to the employer, unless otherwise agreed.<br>5. The protection will be granted to all natural and legal persons who meet the requirements established in this Law for the protection of copyright.
Netherlands	1989	2024	Burgerlijk Wetboek Boek 3 : Vermogensrecht in het algemeen	Art 1. Goederen zijn alle zaken en alle vermogensrechten.<br>Art 2. Zaken zijn de voor menselijke beheersing vatbare stoffelijke objecten.<br>Art 6. Rechten die, hetzij afzonderlijk hetzij tezamen met een ander recht, overdraagbaar zijn, of er toe strekken de rechthebbende stoffelijk voordeel te verschaffen, ofwel verkregen zijn in ruil voor verstrekt of in het vooruitzicht gesteld stoffelijk voordeel, zijn vermogensrechten.
Netherlands	1995	2023	Octrooiwet	Art 2(1). Vatbaar voor octrooi zijn uitvindingen op alle gebieden van de technologie die nieuw zijn, op uitvinderswerkzaamheid berusten en toegepast kunnen worden op het gebied van de nijverheid.<br>Art 2(2). In de zin van het eerste lid worden in het bijzonder niet als uitvindingen beschouwd:<br>a. ontdekkingen, alsmede natuurwetenschappelijke theorieën en wiskundige methoden;<br>b. esthetische vormgevingen;<br>c. stelsels, regels en methoden voor het verrichten van geestelijke arbeid, voor het

				spelen of voor de bedrijfsvoering, alsmede computerprogramma's;<br>d. presentaties van gegevens.
Netherlands	1912	2022	Auteurswet (1/2)	Art 1. Het auteursrecht is het uitsluitend recht van den maker van een werk van letterkunde, wetenschap of kunst, of van diens rechtverkrijgenden, om dit openbaar te maken en te verveelvoudigen, behoudens de beperkingen, bij de wet gesteld.<br>Art 10(1)(12). Onder werken van letterkunde, wetenschap of kunst verstaat deze wet computerprogramma's en het voorbereidend materiaal.<br>Art 13. Onder de verveelvoudiging van een werk van letterkunde, wetenschap of kunst wordt mede verstaan de vertaling, de muziekschikking, de verfilming of tooneelbewerking en in het algemeen iedere geheele of gedeeltelijke bewerking of nabootsing in gewijzigden vorm, welke niet als een nieuw, oorspronkelijk werk moet worden aangemerkt.<br>Art 45i. Onverminderd het bepaalde in Art 13 wordt onder het verveelvoudigen van een werk als bedoeld in Art 10(1)(12), mede verstaan het laden, het in beeld brengen, de uitvoering, de transmissie of de opslag, voor zover voor deze handelingen het verveelvoudigen van dat werk noodzakelijk is.<br>Art 45j. Tenzij anders is overeengekomen, wordt niet als inbreuk op het auteursrecht op een werk als bedoeld in Art 10(1)(12), beschouwd de verveelvoudiging, vervaardigd door de rechtmatige verkrijger van een exemplaar van eerder genoemd werk, die noodzakelijk is voor het met dat werk beoogde gebruik. De verveelvoudiging, als bedoeld in de eerste zin, die geschiedt in het kader van het laden, het in beeld brengen of het verbeteren van fouten, kan niet bij overeenkomst worden verboden.<br>Art 45k. Als inbreuk op het auteursrecht op een werk als bedoeld in Art 10(1)(12), wordt niet beschouwd de verveelvoudiging, vervaardigd door de rechtmatige gebruiker van eerder genoemd werk, die dient als reservekopie indien zulks voor het met dat werk beoogde gebruik noodzakelijk is.
Netherlands	1912	2022	Auteurswet (2/2)	Art 45l. Hij die bevoegd is tot het verrichten van de in artikel 45i bedoelde handelingen, is mede bevoegd tijdens deze handelingen de werking van dat werk waar te nemen, te bestuderen en te testen teneinde de daaraan ten grondslag liggende ideeën en beginselen te achterhalen.<br>Art 45m(1). Als inbreuk op het auteursrecht op een werk als bedoeld in Art 10(1)(12), worden niet beschouwd het vervaardigen van een kopie van dat werk en het vertalen van de codevorm daarvan, indien deze handelingen onmisbaar zijn om de informatie te verkrijgen die nodig is om de interoperabiliteit van een onafhankelijk vervaardigd computerprogramma met andere computerprogramma's tot stand te brengen, mits:<br>a. deze handelingen worden verricht door een persoon die op rechtmatige wijze de beschikking heeft gekregen over een exemplaar van het computerprogramma of door

				een door hem daartoe gemachtigde derde;<br>b. de gegevens die noodzakelijk zijn om de interoperabiliteit tot stand te brengen niet reeds snel en gemakkelijk beschikbaar zijn voor de onder a bedoelde personen;<br>c. deze handelingen beperkt blijven tot die onderdelen van het oorspronkelijke computerprogramma die voor het tot stand brengen van de interoperabiliteit noodzakelijk zijn.<br>Art 45m(2). Het is niet toegestaan de op grond van het eerste lid verkregen informatie:<br>a. te gebruiken voor een ander doel dan het tot stand brengen van de interoperabiliteit van het onafhankelijk vervaardigde computerprogramma;<br>b. aan derden mede te delen, tenzij dit noodzakelijk is voor het tot stand brengen van de interoperabiliteit van het onafhankelijk vervaardigde computerprogramma;<br>c. te gebruiken voor de ontwikkeling, de produktie of het in de handel brengen van een computerprogramma, dat niet als een nieuw, oorspronkelijk werk kan worden aangemerkt of voor andere, op het auteursrecht inbreuk makende handelingen.
Netherlands	2021		Databankenwet (1/3)	Art 1(1). Voor de toepassing van het bij of krachtens deze wet bepaalde wordt verstaan onder:<br>a. databank: een verzameling van werken, gegevens of andere zelfstandige elementen die systematisch of methodisch geordend en afzonderlijk met elektronische middelen of anderszins toegankelijk zijn en waarvan de verkrijging, de controle of de presentatie van de inhoud in kwalitatief of kwantitatief opzicht getuigt van een substantiële investering;<br>b. producent van een databank: degene die het risico draagt van de voor de databank te maken investering;<br>c. opvragen: het permanent of tijdelijk overbrengen van de inhoud van een databank of een deel daarvan op een andere drager, ongeacht op welke wijze en in welke vorm;<br>d. hergebruiken: elke vorm van het aan het publiek ter beschikking stellen van de inhoud van een databank of een deel daarvan door verspreiding van exemplaren, verhuur, on line transmissie of transmissie in een andere vorm<br>Art 1(2). Het voor een beperkte tijd en zonder direct of indirect economisch of commercieel voordeel voor gebruik ter beschikking stellen door voor het publiek toegankelijke instellingen wordt niet als opvragen of hergebruiken beschouwd.<br>Art 2(1). De producent van een databank heeft het uitsluitende recht om toestemming te verlenen voor de volgende handelingen:<br>a. het opvragen of hergebruiken van het geheel of een in kwalitatief of kwantitatief opzicht substantieel deel van de inhoud van de databank;<br>b. het herhaald en systematisch opvragen of hergebruiken van in kwalitatief of in kwantitatief opzicht niet-substantiële delen van de inhoud van een databank, voorzover

				dit in strijd is met de normale exploitatie van die databank of ongerechtvaardigde schade toebrengt aan de rechtmatige belangen van de producent van de databank.<br>Art 2(2). Het auteursrecht of andere rechten op de databank of op de in de databank opgenomen werken, gegevens of andere elementen blijven onverlet.
Netherlands	2021		Databankenwet (2/3)	Art 3(1). De producent van een databank welke op enigerlei wijze aan het publiek ter beschikking is gesteld mag de rechtmatige gebruiker van die databank niet verhinderen in kwalitatief of kwantitatief opzicht niet-substantiële delen van de inhoud ervan op te vragen of te hergebruiken. Voorzover de rechtmatige gebruiker toestemming heeft om slechts een deel van de databank op te vragen of te hergebruiken, geldt de eerste zin slechts voor dat deel.<br>Art 4. De rechtmatige gebruiker van een databank welke op enigerlei wijze aan het publiek ter beschikking is gesteld, mag geen handelingen verrichten waardoor hij de normale exploitatie van de databank in gevaar brengt of ongerechtvaardigde schade aan de producent toebrengt.<br>Art 4a. Als inbreuk op rechten, bedoeld in artikel 2, wordt niet beschouwd:<br>a. opvragingen van een databank, bedoeld in artikel 2, door onderzoeksorganisaties en cultureel erfgoedinstellingen die daartoe rechtmatige toegang hebben, om tekst- en datamining te verrichten met het oog op wetenschappelijk onderzoek;<br>b. onverminderd het in onderdeel a bepaalde, opvragingen van een databank, bedoeld in artikel 2, in het kader van tekst- en datamining door degene die daartoe rechtmatige toegang hebben en het databankenrecht niet uitdrukkelijk op passende wijze is voorbehouden, zoals door middel van machinaal leesbare middelen bij een online ter beschikking gestelde databank;<br>c. opvragingen en hergebruik van een databank, bedoeld in Art 2, uitsluitend ter toelichting bij het onderwijs, voor zover dit door het beoogde, niet commerciële doel wordt gerechtvaardigd;<br>Art 5(1). De rechtmatige gebruiker van een databank die op enigerlei wijze aan het publiek ter beschikking is gesteld mag zonder toestemming van de producent van de databank een substantieel deel van de inhoud van de databank:<br>a. opvragen voor privé doeleinden, mits het een niet-elektronische databank betreft;<br>b. opvragen ter illustratie bij onderwijs of voor wetenschappelijk onderzoek, met bronvermelding en voor zover door het niet-commerciële doel gerechtvaardigd onverminderd het in artikel 4a, onderdelen a en c, bepaalde; en<br>c. opvragen of hergebruiken voor de openbare veiligheid of in het kader van een administratieve of rechterlijke procedure.
Netherlands	2021		Databankenwet (3/3)	Art 6(1). Het recht, bedoeld in artikel 2, eerste lid, ontstaat op het tijdstip waarop de productie van de databank is voltooid. Het vervalt door verloop van vijftien jaar na 1

				januari van het jaar volgend op het tijdstip van voltooiing.<br>Art 6(2). Indien een databank voor het tijdstip waarop de productie werd voltooid ter beschikking van het publiek is gesteld, vervalt het recht, bedoeld in artikel 2, eerste lid, door verloop van vijftien jaar na 1 januari van het jaar volgend op het tijdstip waarop de databank voor het eerst ter beschikking van het publiek werd gesteld.<br>Art 6(3). Met elke in kwalitatief of kwantitatief opzicht substantiële wijziging van de inhoud van de databank, met name door opeenvolgende toevoegingen, weglatingen of veranderingen, die in kwalitatief of kwantitatief opzicht getuigt van een nieuwe substantiële investering, ontstaat een nieuw recht als bedoeld in artikel 2, eerste lid, voor de door die investering ontstane databank.
Netherlands	2018		Wet bescherming bedrijgsgeheimen	Art 1. In deze wet wordt verstaan onder:<br>bedrijfsgeheim: informatie die aan de volgende voorwaarden voldoet:<br>a. zij is geheim in die zin dat zij, in haar geheel dan wel in de juiste samenstelling en ordening van haar bestanddelen, niet algemeen bekend is bij of gemakkelijk toegankelijk is voor degenen binnen de kringen die zich gewoonlijk bezighouden met dergelijke informatie;<br>b. zij bezit handelswaarde omdat zij geheim is, en<br>c. zij is door degene die daar rechtmatig over beschikt, onderworpen aan redelijke maatregelen, gezien de omstandigheden, om deze geheim te houden;<br>houder van het bedrijfsgeheim: iedere natuurlijke persoon of rechtspersoon die rechtmatig over een bedrijfsgeheim beschikt;<br>inbreukmakende goederen: goederen waarvan het ontwerp, de kenmerken, de werking, het productieproces of het in de handel brengen aanzienlijk voordeel heeft of hebben bij bedrijfsgeheimen die onrechtmatig zijn verkregen, gebruikt of openbaar gemaakt;<br>inbreukmaker: iedere natuurlijke persoon of rechtspersoon die een bedrijfsgeheim onrechtmatig heeft verkregen, gebruikt of openbaar gemaakt.<br>Art 2(1). Het verkrijgen van een bedrijfsgeheim zonder de toestemming van de houder van het bedrijfsgeheim is onrechtmatig wanneer het bedrijfsgeheim is verkregen door middel van:<br>a. onbevoegde toegang tot of het zich onbevoegd toe-eigenen of kopiëren van documenten, voorwerpen, substanties, materialen of elektronische bestanden waarover de houder van het bedrijfsgeheim rechtmatig beschikt en die het bedrijfsgeheim bevatten of waaruit het bedrijfsgeheim kan worden afgeleid;<br>b. andere gedragingen die, gezien de omstandigheden, worden beschouwd als strijdig met eerlijke handelspraktijken.
Netherlands	2018		Wet bescherming bedrijgsgeheimen	Art 2(2). Het gebruiken of openbaar maken van een bedrijfsgeheim is onrechtmatig wanneer het bedrijfsgeheim zonder de toestemming van de houder van het

				bedrijfsgeheim wordt gebruikt of openbaar gemaakt door een natuurlijke persoon of rechtspersoon die:<br>a. het bedrijfsgeheim op onrechtmatige wijze heeft verkregen;<br>b. inbreuk maakt op een geheimhoudingsovereenkomst of een andere verplichting tot het niet openbaar maken van het bedrijfsgeheim, of<br>c. inbreuk maakt op een contractuele of andere verplichting tot beperking van het gebruik van het bedrijfsgeheim.<br>Art 2(3). Het verkrijgen, gebruiken of openbaar maken van een bedrijfsgeheim is ook onrechtmatig wanneer een natuurlijke persoon of rechtspersoon op het moment van het verkrijgen, gebruiken, of openbaar maken, wist, of gezien de omstandigheden, had moeten weten dat het bedrijfsgeheim direct of indirect werd verkregen van een andere natuurlijke persoon of rechtspersoon die het bedrijfsgeheim op een onrechtmatige manier gebruikte of openbaar maakte als bedoeld in het tweede lid.<br>Art 2(4). Het produceren, aanbieden of in de handel brengen van inbreukmakende goederen, of de invoer, uitvoer of opslag van inbreukmakende goederen voor die doeleinden, wordt ook als een onrechtmatig gebruik van een bedrijfsgeheim beschouwd wanneer de natuurlijke persoon of rechtspersoon die dergelijke activiteiten uitvoert, wist of, gezien de omstandigheden, had moeten weten dat het bedrijfsgeheim onrechtmatig werd gebruikt.
Netherlands	2018		Wet bescherming bedrijgsgeheimen	Art 3(1). Als het onrechtmatig verkrijgen van een bedrijfsgeheim wordt niet beschouwd het verkrijgen door middel van:<br>a. onafhankelijke ontdekking of onafhankelijk ontwerp;<br>b. observatie, onderzoek, demontage of testen van een product of voorwerp dat ter beschikking van het publiek is gesteld of dat op een rechtmatige manier in het bezit is van degene die de informatie verwerft en die niet gebonden is aan een rechtsgeldige verplichting het verkrijgen van het bedrijfsgeheim te beperken;<br>c. uitoefening van het recht van werknemers of vertegenwoordigers daarvan op informatie en raadpleging in overeenstemming met het recht van de Europese Unie of met bepalingen bij of krachtens de wet of met nationale praktijken, of<br>d. iedere andere praktijk die, gezien de omstandigheden, in overeenstemming is met eerlijke handelspraktijken.<br>Art 3(2). Als het onrechtmatig verkrijgen, gebruiken of openbaar maken van een bedrijfsgeheim wordt niet beschouwd het verkrijgen, gebruiken of openbaar maken dat op grond van het recht van de Europese Unie of bepalingen bij of krachtens de wet vereist of toegestaan is.
Germany			Markengesetz	§ 1 Geschützte Marken und sonstige Kennzeichen<br>Nach diesem Gesetz werden geschützt: 1.Marken, 2.geschäftliche Bezeichnungen,

				3.geographische Herkunftsangaben.<br><br>§ 3 Als Marke schutzfähige Zeichen<br>(1) Als Marke können alle Zeichen, insbesondere Wörter einschließlich Personennamen, Abbildungen, Buchstaben, Zahlen, Klänge, dreidimensionale Gestaltungen einschließlich der Form einer Ware oder ihrer Verpackung sowie sonstige<br>Aufmachungen einschließlich Farben und Farbzusammenstellungen geschützt werden, die geeignet sind, Waren oder Dienstleistungen eines Unternehmens von denjenigen anderer Unternehmen zu unterscheiden.<br>(2) Dem Markenschutz nicht zugänglich sind Zeichen, die ausschließlich aus Formen oder anderen charakteristischen Merkmalen bestehen, 1. die durch die Art der Ware selbst bedingt sind, 2. die zur Erreichung einer technischen Wirkung erforderlich sind oder 3. die der Ware einen wesentlichen Wert verleihen.<br><br>§ 7 Inhaberschaft<br>Inhaber von eingetragenen und angemeldeten Marken können sein: 1.natürliche Personen,2.juristische Personen oder3.Personengesellschaften, sofern sie mit der Fähigkeit ausgestattet sind, Rechte zu erwerben und Verbindlichkeiten einzugehen.
Germany			Patentgesetz	§ 1<br>(1) Patente werden für Erfindungen auf allen Gebieten der Technik erteilt, sofern sie neu sind, auf einer erfinderischen Tätigkeit beruhen und gewerblich anwendbar sind.<br>(3) Als Erfindungen im Sinne des Absatzes 1 werden insbesondere nicht angesehen:<br>1. Entdeckungen sowie wissenschaftliche Theorien und mathematische Methoden;<br>2. ästhetische Formschöpfungen;<br>3. Pläne, Regeln und Verfahren für gedankliche Tätigkeiten, für Spiele oder für geschäftliche Tätigkeiten sowie Programme für Datenverarbeitungsanlagen;<br>4. die Wiedergabe von Informationen.
			Patentgesetz	§ 2<br>(1) Für Erfindungen, deren gewerbliche Verwertung gegen die öffentliche Ordnung oder die guten Sitten verstoßen würde, werden keine Patente erteilt; ein solcher Verstoß kann nicht allein aus der Tatsache hergeleitet werden, dass die Verwertung durch Gesetz oder Verwaltungsvorschrift verboten ist.
			Patentgesetz	§ 13<br>(1) Die Wirkung des Patents tritt insoweit nicht ein, als die Bundesregierung anordnet, daß die Erfindung im Interesse der öffentlichen Wohlfahrt benutzt werden soll. Sie erstreckt sich ferner nicht auf eine Benutzung der Erfindung, die im Interesse der

				Sicherheit des Bundes von der zuständigen obersten Bundesbehörde oder in deren Auftrag von einer nachgeordneten Stelle angeordnet wird
			Patentgesetz	§ 21<br>(1) Das Patent wird widerrufen (§ 61), wenn sich ergibt, daß<br>1. der Gegenstand des Patents nach den §§ 1 bis 5 nicht patentfähig ist,<br>2. das Patent die Erfindung nicht so deutlich und vollständig offenbart, daß ein Fachmann sie ausführen kann,<br>3.der wesentliche Inhalt des Patents den Beschreibungen, Zeichnungen, Modellen, Gerätschaften oder Einrichtungen eines anderen oder einem von diesem angewendeten Verfahren ohne dessen Einwilligung entnommen worden ist (widerrechtliche Entnahme),<br>4.der Gegenstand des Patents über den Inhalt der Anmeldung in der Fassung hinausgeht, in der sie bei der für die Einreichung der Anmeldung zuständigen Behörde ursprünglich eingereicht worden ist; das gleiche gilt, wenn das Patent auf einer Teilanmeldung oder einer nach § 7 Abs. 2 eingereichten neuen Anmeldung beruht und der Gegenstand des Patents über den Inhalt der früheren Anmeldung in der Fassung hinausgeht, in der sie bei der für die Einreichung der früheren Anmeldung zuständigen Behörde ursprünglich eingereicht worden ist.
Germany			Urhebergesetz	§ 1 Allgemeines<br>Die Urheber von Werken der Literatur, Wissenschaft und Kunst genießen für ihre Werke Schutz nach Maßgabe dieses Gesetzes.
			Urhebergesetz	§ 2 Geschützte Werke<br>(1) Zu den geschützten Werken der Literatur, Wissenschaft und Kunst gehören insbesondere:<br>1. Sprachwerke, wie Schriftwerke, Reden und Computerprogramme;<br>2. Werke der Musik;<br>3. pantomimische Werke einschließlich der Werke der Tanzkunst;<br>4. Werke der bildenden Künste einschließlich der Werke der Baukunst und der angewandten Kunst und Entwürfe solcher Werke;<br>5. Lichtbildwerke einschließlich der Werke, die ähnlich wie Lichtbildwerke geschaffen werden;<br>6. Filmwerke einschließlich der Werke, die ähnlich wie Filmwerke geschaffen werden;<br>7. Darstellungen wissenschaftlicher oder technischer Art, wie Zeichnungen, Pläne, Karten, Skizzen, Tabellen und plastische Darstellungen.<br>(2) Werke im Sinne dieses Gesetzes sind nur persönliche geistige Schöpfungen.
			Urhebergesetz	§ 4 Sammelwerke und Datenbankwerke<br>(1) Sammlungen von Werken, Daten oder anderen unabhängigen Elementen, die

				aufgrund der Auswahl oder Anordnung der Elemente eine persönliche geistige Schöpfung sind (Sammelwerke), werden, unbeschadet eines an den einzelnen Elementen gegebenenfalls bestehenden Urheberrechts oder verwandten Schutzrechts, wie selbständige Werke geschützt.<br>(2) Datenbankwerk im Sinne dieses Gesetzes ist ein Sammelwerk, dessen Elemente systematisch oder methodisch angeordnet und einzeln mit Hilfe elektronischer Mittel oder auf andere Weise zugänglich sind. Ein zur Schaffung des Datenbankwerkes oder zur Ermöglichung des Zugangs zu dessen Elementen verwendetes Computerprogramm (§ 69a) ist nicht Bestandteil des Datenbankwerkes.
			Urhebergesetz	§ 15 Allgemeines<br>(1) Der Urheber hat das ausschließliche Recht, sein Werk in körperlicher Form zu verwerten; das Recht umfaßt insbesondere<br>1.das Vervielfältigungsrecht (§ 16),<br>2.das Verbreitungsrecht (§ 17),<br>3.das Ausstellungsrecht (§ 18).
			Urhebergesetz	§ 23 Bearbeitungen und Umgestaltungen<br>(1) Bearbeitungen oder andere Umgestaltungen eines Werkes, insbesondere auch einer Melodie, dürfen nur mit Zustimmung des Urhebers veröffentlicht oder verwertet werden. Wahrt das neu geschaffene Werk einen hinreichenden Abstand zum benutzten Werk, so liegt keine Bearbeitung oder Umgestaltung im Sinne des Satzes 1 vor.
			Urhebergesetz	§ 44b Text und Data Mining<br>(1) Text und Data Mining ist die automatisierte Analyse von einzelnen oder mehreren digitalen oder digitalisierten Werken, um daraus Informationen insbesondere über Muster, Trends und Korrelationen zu gewinnen.<br>(2) Zulässig sind Vervielfältigungen von rechtmäßig zugänglichen Werken für das Text und Data Mining. Die Vervielfältigungen sind zu löschen, wenn sie für das Text und Data Mining nicht mehr erforderlich sind.<br>(3) Nutzungen nach Absatz 2 Satz 1 sind nur zulässig, wenn der Rechtsinhaber sich diese nicht vorbehalten hat. Ein Nutzungsvorbehalt bei online zugänglichen Werken ist nur dann wirksam, wenn er in maschinenlesbarer Form erfolgt.
			Urhebergesetz	§ 60c Wissenschaftliche Forschung<br>(1) Zum Zweck der nicht kommerziellen wissenschaftlichen Forschung dürfen bis zu 15 Prozent eines Werkes vervielfältigt, verbreitet und öffentlich zugänglich gemacht werden<br>1. für einen bestimmt abgegrenzten Kreis von Personen für deren eigene wissenschaftliche Forschung sowie<br>2. für einzelne Dritte, soweit dies der Überprüfung der Qualität wissenschaftlicher

				Forschung dient.<br>(2) Für die eigene wissenschaftliche Forschung dürfen bis zu 75 Prozent eines Werkes vervielfältigt werden.<br>(3) Abbildungen, einzelne Beiträge aus derselben Fachzeitschrift oder wissenschaftlichen Zeitschrift, sonstige Werke geringen Umfangs und vergriffene Werke dürfen abweichend von den Absätzen 1 und 2 vollständig genutzt werden.<br>(4) Nicht nach den Absätzen 1 bis 3 erlaubt ist es, während öffentlicher Vorträge, Aufführungen oder Vorführungen eines Werkes diese auf Bild- oder Tonträger aufzunehmen und später öffentlich zugänglich zu machen.
			Urhebergesetz	60d Text und Data Mining für Zwecke der wissenschaftlichen Forschung<br>(1) Vervielfältigungen für Text und Data Mining (§ 44b Absatz 1 und 2 Satz 1) sind für Zwecke der wissenschaftlichen Forschung nach Maßgabe der nachfolgenden Bestimmungen zulässig.<br>(2) Zu Vervielfältigungen berechtigt sind Forschungsorganisationen. Forschungsorganisationen sind Hochschulen, Forschungsinstitute oder sonstige Einrichtungen, die wissenschaftliche Forschung betreiben, sofern sie<br>1. nicht kommerzielle Zwecke verfolgen,<br>2. sämtliche Gewinne in die wissenschaftliche Forschung reinvestieren oder<br>3. im Rahmen eines staatlich anerkannten Auftrags im öffentlichen Interesse tätig sind.<br>Nicht nach Satz 1 berechtigt sind Forschungsorganisationen, die mit einem privaten Unternehmen zusammenarbeiten, das einen bestimmenden Einfluss auf die Forschungsorganisation und einen bevorzugten Zugang zu den Ergebnissen der wissenschaftlichen Forschung hat.<br>(3) Zu Vervielfältigungen berechtigt sind ferner<br>1. Bibliotheken und Museen, sofern sie öffentlich zugänglich sind, sowie Archive und Einrichtungen im Bereich des Film- oder Tonerbes (Kulturerbe-Einrichtungen),<br>2. einzelne Forscher, sofern sie nicht kommerzielle Zwecke verfolgen.<br>(4) Berechtigte nach den Absätzen 2 und 3, die nicht kommerzielle Zwecke verfolgen, dürfen Vervielfältigungen nach Absatz 1 folgenden Personen öffentlich zugänglich machen:<br>1. einem bestimmt abgegrenzten Kreis von Personen für deren gemeinsame wissenschaftliche Forschung sowie<br>2. einzelnen Dritten zur Überprüfung der Qualität wissenschaftlicher Forschung.<br>Sobald die gemeinsame wissenschaftliche Forschung oder die Überprüfung der Qualität wissenschaftlicher Forschung abgeschlossen ist, ist die öffentliche Zugänglichmachung zu beenden.

				(5) Berechtigte nach den Absätzen 2 und 3 Nummer 1 dürfen Vervielfältigungen nach Absatz 1 mit angemessenen Sicherheitsvorkehrungen gegen unbefugte Benutzung aufbewahren, solange sie für Zwecke der wissenschaftlichen Forschung oder zur Überprüfung wissenschaftlicher Erkenntnisse erforderlich sind.<br>(6) Rechtsinhaber sind befugt, erforderliche Maßnahmen zu ergreifen, um zu verhindern, dass die Sicherheit und Integrität ihrer Netze und Datenbanken durch Vervielfältigungen nach Absatz 1 gefährdet werden.
			Urhebergesetz	Abschnitt 8 - Besondere Bestimmungen für Computerprogramme<br>§ 69a Gegenstand des Schutzes<br>(1) Computerprogramme im Sinne dieses Gesetzes sind Programme in jeder Gestalt, einschließlich des Entwurfsmaterials.<br>(2) Der gewährte Schutz gilt für alle Ausdrucksformen eines Computerprogramms. Ideen und Grundsätze, die einem Element eines Computerprogramms zugrunde liegen, einschließlich der den Schnittstellen zugrundeliegenden Ideen und Grundsätze, sind nicht geschützt.<br>(3) Computerprogramme werden geschützt, wenn sie individuelle Werke in dem Sinne darstellen, daß sie das Ergebnis der eigenen geistigen Schöpfung ihres Urhebers sind. Zur Bestimmung ihrer Schutzfähigkeit sind keine anderen Kriterien, insbesondere nicht qualitative oder ästhetische, anzuwenden.
			Urhebergesetz	§ 69b Urheber in Arbeits- und Dienstverhältnissen<br>(1) Wird ein Computerprogramm von einem Arbeitnehmer in Wahrnehmung seiner Aufgaben oder nach den Anweisungen seines Arbeitgebers geschaffen, so ist ausschließlich der Arbeitgeber zur Ausübung aller vermögensrechtlichen Befugnisse an dem Computerprogramm berechtigt, sofern nichts anderes vereinbart ist.<br>(2) Absatz 1 ist auf Dienstverhältnisse entsprechend anzuwenden.
Germany			Verordnung über ergänzende Bestimmungen zur Nutzung nicht verfügbarer Werke nach dem Urheberrechtsgesetz und dem Verwertungsgesellschaftengesetz (Nicht-verfügbare-Werke-Verordnung — NvWV)	§ 3 Wahrung des Urheberpersönlichkeitsrechts bei nicht veröffentlichten Werken<br>(1) Bei nicht veröffentlichten Werken ist im Wege der Einzelfallabwägung zu prüfen, ob das Informationsinteresse der Allgemeinheit an der Nutzung des Werkes den durch die Veröffentlichung bewirkten Eingriff in das Urheberpersönlichkeitsrecht rechtfertigt. Hierbei sind insbesondere die Schöpfungshöhe und der Persönlichkeitsrechtsbezug des Werkes zu berücksichtigen.<br>(2) Nach Ablauf der in § 11 des Bundesarchivgesetzes bestimmten Schutzfristen wird das Überwiegen des Informationsinteresses widerleglich vermutet. Dies gilt insbesondere für Werke in Archivgut von Behörden und Gerichten.<br>(3) Die Vermutung nach Absatz 2 ist insbesondere dann widerlegt, wenn konkrete Anhaltspunkte dafür bestehen, dass der Urheber von einer Veröffentlichung des Werkes absehen wollte.

Germany			Gesetz zum Schutz von Geschäftsgeheimnissen (GeschGehG)	§ 1 Anwendungsbereich<br>(1) Dieses Gesetz dient dem Schutz von Geschäftsgeheimnissen vor unerlaubter Erlangung, Nutzung und Offenlegung.
Germany				§ 2 Begriffsbestimmungen<br>Im Sinne dieses Gesetzes ist<br><br>1. Geschäftsgeheimnis<br>eine Information<br>a)die weder insgesamt noch in der genauen Anordnung und Zusammensetzung ihrer Bestandteile den Personen in den Kreisen, die üblicherweise mit dieser Art von Informationen umgehen, allgemein bekannt oder ohne Weiteres zugänglich ist und daher von wirtschaftlichem Wert ist und<br>b)die Gegenstand von den Umständen nach angemessenen Geheimhaltungsmaßnahmen durch ihren rechtmäßigen Inhaber ist und<br>c) bei der ein berechtigtes Interesse an der Geheimhaltung besteht;<br><br>2.Inhaber eines Geschäftsgeheimnisses<br>jede natürliche oder juristische Person, die die rechtmäßige Kontrolle über ein Geschäftsgeheimnis hat;<br><br>3.Rechtsverletzer<br>jede natürliche oder juristische Person, die entgegen § 4 ein Geschäftsgeheimnis rechtswidrig erlangt, nutzt oder offenlegt; Rechtsverletzer ist nicht, wer sich auf eine Ausnahme nach § 5 berufen kann;<br><br>4.rechtsverletzendes Produkt<br>ein Produkt, dessen Konzeption, Merkmale, Funktionsweise, Herstellungsprozess oder Marketing in erheblichem Umfang auf einem rechtswidrig erlangten, genutzten oder offengelegten Geschäftsgeheimnis beruht.
Germany				§ 5 Ausnahmen<br>Die Erlangung, die Nutzung oder die Offenlegung eines Geschäftsgeheimnisses fällt nicht unter die Verbote des § 4, wenn dies zum Schutz eines berechtigten Interesses erfolgt, insbesondere<br><br>1.zur Ausübung des Rechts der freien Meinungsäußerung und der Informationsfreiheit, einschließlich der Achtung der Freiheit und der Pluralität der Medien;<br>2.zur Aufdeckung einer rechtswidrigen Handlung oder eines beruflichen oder

				sonstigen Fehlverhaltens, wenn die Erlangung, Nutzung oder Offenlegung geeignet ist, das allgemeine öffentliche Interesse zu schützen;<br>3.im Rahmen der Offenlegung durch Arbeitnehmer gegenüber der Arbeitnehmervertretung, wenn dies erforderlich ist, damit die Arbeitnehmervertretung ihre Aufgaben erfüllen kann.
United Kingdom	1977	2024		Section 1 Patentable inventions.<br>(1)A patent may be granted only for an invention in respect of which the following conditions are satisfied, that is to say—<br><br>(a)the invention is new;<br><br>(b)it involves an inventive step;<br><br>(c)it is capable of industrial application;<br><br>(d)the grant of a patent for it is not excluded by subsections (2) and (3) [F1 or section 4A] below;<br><br>and references in this Act to a patentable invention shall be construed accordingly.<br>(2)It is hereby declared that the following (among other things) are not inventions for the purposes of this Act, that is to say, anything which consists of—<br><br>(a)a discovery, scientific theory or mathematical method;<br><br>(b)a literary, dramatic, musical or artistic work or any other aesthetic creation whatsoever;<br><br>(c)a scheme, rule or method for performing a mental act, playing a game or doing business, or a program for a computer;<br><br>(d)the presentation of information;<br><br>but the foregoing provision shall prevent anything from being treated as an invention for the purposes of this Act only to the extent that a patent or application for a patent relates to that thing as such.<br>[F2(3)A patent shall not be granted for an invention the commercial exploitation of which would be contrary to public policy or morality.

				(4)For the purposes of subsection (3) above exploitation shall not be regarded as contrary to public policy or morality only because it is prohibited by any law in force in the United Kingdom or any part of it.]<br><br>(5)The Secretary of State may by order vary the provisions of subsection (2) above for the purpose of maintaining them in conformity with developments in science and technology; and no such order shall be made unless a draft of the order has been laid before, and approved by resolution of, each House of Parliament.<br><br>Section 7 Right to apply for and obtain a patent.<br>(1)Any person may make an application for a patent either alone or jointly with another.<br><br>(2)A patent for an invention may be granted—<br><br>(a)primarily to the inventor or joint inventors;<br><br>(b)in preference to the foregoing, to any person or persons who, by virtue of any enactment or rule of law, or any foreign law or treaty or international convention, or by virtue of an enforceable term of any agreement entered into with the inventor before the making of the invention, was or were at the time of the making of the invention entitled to the whole of the property in it (other than equitable interests) in the United Kingdom;<br><br>(c)in any event, to the successor or successors in title of any person or persons mentioned in paragraph (a) or (b) above or any person so mentioned and the successor or successors in title of another person so mentioned;<br><br>and to no other person.<br>(3)In this Act “inventor” in relation to an invention means the actual deviser of the invention and “joint inventor” shall be construed accordingly.<br><br>(4)Except so far as the contrary is established, a person who makes an application for a patent shall be taken to be the person who is entitled under subsection (2) above to be granted a patent and two or more persons who make such an application jointly shall be taken to be the persons so entitled.

				8 Determination before grant of questions about entitlement to patents, etc.<br>(1)At any time before a patent has been granted for an invention (whether or not an application has been made for it)—<br><br>(a)any person may refer to the comptroller the question whether he is entitled to be granted (alone or with any other persons) a patent for that invention or has or would have any right in or under any patent so granted or any application for such a patent; or<br><br>(b)any of two or more co-proprietors of an application for a patent for that invention may so refer the question whether any right in or under the application should be transferred or granted to any other person;<br><br>and the comptroller shall determine the question and may make such order as he thinks fit to give effect to the determination.<br>(2)Where a person refers a question relating to an invention under subsection (1)(a) above to the comptroller after an application for a patent for the invention has been filed and before a patent is granted in pursuance of the application, then, unless the application is refused or withdrawn before the reference is disposed of by the comptroller, the comptroller may, without prejudice to the generality of subsection (1) above and subject to subsection (6) below.—<br><br>(a)order that the application shall proceed in the name of that person, either solely or jointly with that of any other applicant, instead of in the name of the applicant or any specified applicant;<br><br>(b)where the reference was made by two or more persons, order that the application shall proceed in all their names jointly;<br><br>(c)refuse to grant a patent in pursuance of the application or order the application to be amended so as to exclude any of the matter in respect of which the question was referred;<br><br>(d)make an order transferring or granting any licence or other right in or under the application and give directions to any person for carrying out the provisions of any such order.

				(3)Where a question is referred to the comptroller under subsection (1)(a) above and—<br><br>(a)the comptroller orders an application for a patent for the invention to which the question relates to be so amended;<br><br>(b)any such application is refused under subsection 2(c) above before the comptroller has disposed of the reference (whether the reference was made before or after the publication of the application); or<br><br>(c)any such application is refused under any other provision of this Act or is withdrawn before the comptroller has disposed of the reference [F1(whether the application is refused or withdrawn before or after its publication)] ;<br><br>the comptroller may order that any person by whom the reference was made may within the prescribed period make a new application for a patent for the whole or part of any matter comprised in the earlier application or, as the case may be, for all or any of the matter excluded from the earlier application, subject in either case to section 76 below, and in either case that, if such a new application is made, it shall be treated as having been filed on the date of filing the earlier application.<br>(4)Where a person refers a question under subsection (1)(b) above relating to an application, any order under subsection (1) above may contain directions to any person for transferring or granting any right in or under the application.<br><br>(5)If any person to whom directions have been given under subsection (2)(d) or (4) above fails to do anything necessary for carrying out any such directions within 14 days after the date of the directions, the comptroller may, on application made to him by any person in whose favour or on whose reference the directions were given, authorise him to do that thing on behalf of the person to whom the directions were given.<br><br>(6)Where on a reference under this section it is alleged that, by virtue of any transaction, instrument or event relating to an invention or an application for a patent, any person other than the inventor or the applicant for the patent has become entitled to be granted (whether alone or with any other persons) a patent for the invention or has or would have any right in or under any patent so granted or any application for any such patent, an order shall not be made under subsection (2)(a), (b) or (d) above on the reference unless notice of the reference is given to the applicant and any such person,

				except any of them who is a party to the reference.<br><br>(7)If it appears to the comptroller on a reference of a question under this section that the question involves matters which would more properly be determined by the court, he may decline to deal with it and, without prejudice to the court's jurisdiction to determine any such question and make a declaration, or any declaratory jurisdiction of the court in Scotland, the court shall have jurisdiction to do so.<br><br>(8)No directions shall be given under this section so as to affect the mutual rights or obligations of trustees or of the personal representatives of deceased persons, or their right or obligations as such.
United Kingdom	2023		Thaler (Appellant) v Comptroller-General of Patents, Designs and Trade Marks (Respondent)	The structure and content of sections 7 and 13 of the Act, on their own and in the context of the Act as a whole, permit only one interpretation: an inventor within the meaning of the 1977 Act must be a natural person, and DABUS is not a person at all, let alone a natural person: it is a machine and on the factual assumption underpinning these proceedings, created or generated the technical advances disclosed in the applications on its own.
United Kingdom	1988	2024		Section 9 Authorship of work.<br>(1)In this Part "author", in relation to a work, means the person who creates it.<br><br>(2)That person shall be taken to be—<br><br>F1 [F1(aa)in the case of a sound recording, the producer;<br><br>F1(ab)in the case of a film, the producer and the principal director;]<br><br>(b)in the case of a broadcast, the person making the broadcast (see section 6(3)) or, in the case of a broadcast which relays another broadcast by reception and immediate re-transmission, the person making that other broadcast;<br><br>(c)F2. . . . .<br><br>(d)in the case of the typographical arrangement of a published edition, the publisher.<br><br>(3)In the case of a literary, dramatic, musical or artistic work which is computer-generated, the author shall be taken to be the person by whom the arrangements necessary for the creation of the work are undertaken.

				(4)For the purposes of this Part a work is of “unknown authorship” if the identity of the author is unknown or, in the case of a work of joint authorship, if the identity of none of the authors is known.<br><br>(5)For the purposes of this Part the identity of an author shall be regarded as unknown if it is not possible for a person to ascertain his identity by reasonable inquiry; but if his identity is once known it shall not subsequently be regarded as unknown.
United Kingdom	1994	2020		Section 10 Infringement of registered trade mark.<br>(1)A person infringes a registered trade mark if he uses in the course of trade a sign which is identical with the trade mark in relation to goods or services which are identical with those for which it is registered.<br><br>(2)A person infringes a registered trade mark if he uses in the course of trade a sign where because—<br><br>(a)the sign is identical with the trade mark and is used in relation to goods or services similar to those for which the trade mark is registered, or<br><br>(b)the sign is similar to the trade mark and is used in relation to goods or services identical with or similar to those for which the trade mark is registered,<br><br>there exists a likelihood of confusion on the part of the public, which includes the likelihood of association with the trade mark.<br>(3)A person infringes a registered trade mark if he uses in the course of trade[F1, in relation to goods or services,] a sign which—<br><br>(a)is identical with or similar to the trade mark, F2...<br><br>F2(b). . . . .<br><br>where the trade mark has a reputation in the United Kingdom and the use of the sign, being without due cause, takes unfair advantage of, or is detrimental to, the distinctive character or the repute of the trade mark.<br>[F3(3A)Subsection (3) applies irrespective of whether the goods and services in relation to which the sign is used are identical with, similar to or not similar to those for which the trade mark is registered.

				(3B)Where the risk exists that the packaging, labels, tags, security or authenticity features or devices, or any other means to which the trade mark is affixed could be used in relation to goods or services and that use would constitute an infringement of the rights of the proprietor of the trade mark, a person infringes a registered trade mark if the person carries out in the course of trade any of the following acts—<br><br>(a)affixing a sign identical with, or similar to, the trade mark on packaging, labels, tags, security or authenticity features or devices, or any other means to which the mark may be affixed; or<br><br>(b)offering or placing on the market, or stocking for those purposes, or importing or exporting, packaging, labels, tags, security or authenticity features or devices, or any other means to which the mark is affixed.]<br><br>(4)For the purposes of this section a person uses a sign if, in particular, he—<br><br>(a)affixes it to goods or the packaging thereof;<br><br>(b)offers or exposes goods for sale, puts them on the market or stocks them for those purposes under the sign, or offers or supplies services under the sign;<br><br>(c)imports or exports goods under the sign;F4...<br><br>[F5(ca)uses the sign as a trade or company name or part of a trade or company name;]<br><br>(d)uses the sign on business papers [F6and] in advertising[F7; or]<br><br>[F8(e)uses the sign in comparative advertising in a manner that is contrary to the Business Protection from Misleading Marketing Regulations 2008.]<br><br>F9(5). . . . .<br><br>F9(6). . . . .
United Kingdom	2014			Research, private study and text and data analysis for non-commercial research<br>3.—(1) In section 29(1)—

				(a)in subsection (1), omit “literary, dramatic, musical or artistic”,<br><br>(b)in subsection (1C), omit “literary, dramatic, musical or artistic”,<br><br>(c)omit subsection (2),<br><br>(d)for subsection (3)(a), substitute—<br><br>“(a)in the case of a librarian, or a person acting on behalf of a librarian, that person does anything which is not permitted under section 42A (copying by librarians: single copies of published works), or”, and<br><br>(e)after subsection (4A) insert—<br><br>“(4B) To the extent that a term of a contract purports to prevent or restrict the doing of any act which, by virtue of this section, would not infringe copyright, that term is unenforceable.”.<br><br>(2) After section 29 insert—<br><br>“29A Copies for text and data analysis for non-commercial research<br>(1) The making of a copy of a work by a person who has lawful access to the work does not infringe copyright in the work provided that—<br><br>(a)the copy is made in order that a person who has lawful access to the work may carry out a computational analysis of anything recorded in the work for the sole purpose of research for a non-commercial purpose, and<br><br>(b)the copy is accompanied by a sufficient acknowledgement (unless this would be impossible for reasons of practicality or otherwise).<br><br>(2) Where a copy of a work has been made under this section, copyright in the work is infringed if—<br><br>(a)the copy is transferred to any other person, except where the transfer is authorised by the copyright owner, or

				(b)the copy is used for any purpose other than that mentioned in subsection (1)(a), except where the use is authorised by the copyright owner.<br><br>(3) If a copy made under this section is subsequently dealt with—<br><br>(a)it is to be treated as an infringing copy for the purposes of that dealing, and<br><br>(b)if that dealing infringes copyright, it is to be treated as an infringing copy for all subsequent purposes.<br><br>(4) In subsection (3) "dealt with" means sold or let for hire, or offered or exposed for sale or hire.<br><br>(5) To the extent that a term of a contract purports to prevent or restrict the making of a copy which, by virtue of this section, would not infringe copyright, that term is unenforceable.".<br><br>(3) In Schedule 2(2), immediately before paragraph 2, insert—<br><br>"Research and private study<br><br>1C.—(1) Fair dealing with a performance or a recording of a performance for the purposes of research for a non-commercial purpose does not infringe the rights conferred by this Chapter.<br><br>(2) Fair dealing with a performance or recording of a performance for the purposes of private study does not infringe the rights conferred by this Chapter.<br><br>(3) Copying of a recording by a person other than the researcher or student is not fair dealing if—<br><br>(a)in the case of a librarian, or a person acting on behalf of a librarian, that person does anything which is not permitted under paragraph 6F (copying by librarians: single copies of published recordings), or<br><br>(b)in any other case, the person doing the copying knows or has reason to believe that it will result in copies of substantially the same material being provided to more than

				one person at substantially the same time and for substantially the same purpose.<br><br>(4) To the extent that a term of a contract purports to prevent or restrict the doing of any act which, by virtue of this paragraph, would not infringe any right conferred by this Chapter, that term is unenforceable.<br><br>(5) Expressions used in this paragraph have the same meaning as in section 29.<br><br>Copies for text and data analysis for non-commercial research<br><br>1D.—(1) The making of a copy of a recording of a performance by a person who has lawful access to the recording does not infringe any rights conferred by this Chapter provided that the copy is made in order that a person who has lawful access to the recording may carry out a computational analysis of anything recorded in the recording for the sole purpose of research for a non-commercial purpose.<br><br>(2) Where a copy of a recording has been made under this paragraph, the rights conferred by this Chapter are infringed if—<br><br>(a)the copy is transferred to any other person, except where the transfer is authorised by the rights owner, or<br><br>(b)the copy is used for any purpose other than that mentioned in sub-paragraph (1), except where the use is authorised by the rights owner.<br><br>(3) If a copy of a recording made under this paragraph is subsequently dealt with—<br><br>(a)it is to be treated as an illicit recording for the purposes of that dealing, and<br><br>(b)if that dealing infringes any right conferred by this Chapter, it is to be treated as an illicit recording for all subsequent purposes.<br><br>(4) To the extent that a term of a contract purports to prevent or restrict the making of a copy which, by virtue of this paragraph, would not infringe any right conferred by this Chapter, that term is unenforceable.<br><br>(5) Expressions used in this paragraph have the same meaning as in section 29A.”.

United Kingdom	2021	2022		Schedule 3: Interpretation<br>1. In this Schedule—<br><br>“artificial intelligence” means technology enabling the programming or training of a device or software to—<br>(i)<br>perceive environments through the use of data;<br><br>(ii)<br>interpret data using automated processing designed to approximate cognitive abilities; and<br><br>(iii)<br>make recommendations, predictions or decisions;<br><br>with a view to achieving a specific objective;<br>“advanced robotics” has the same meaning as in Schedule 2;<br>“cognitive abilities” means reasoning, perception, communication, learning, planning, problem solving, abstract thinking, decision-making or organisation;<br>“cyber security” means the activities necessary to protect network and information systems, the users of such systems, and other persons affected by cyber threats;<br>“cyber threat” means any potential circumstance, event or action that could damage, disrupt or otherwise adversely affect network and information systems, the users of such systems and other persons;<br>“network and information system” has the same meaning as in regulation 1 of the Network and Information Systems Regulations 2018(1);<br>“technology” has the same meaning as in Schedule 2 to the Export Control Order 2008(2).<br>Commencement Information<br><br>I1 Sch. 3 para. 1 in force at 4.1.2022, see reg. 1(2)<br>Activities – artificial intelligence<br>2.—(1) A qualifying entity carrying on any of the following activities for one or more of the purposes set out in sub-paragraph 2—<br><br>(a)research into artificial intelligence; or

				(b)developing or producing goods, software or technology that use artificial intelligence.<br><br>(2) The purposes are—<br><br>(a)the identification or tracking of objects, people or events;<br><br>(b)advanced robotics;<br><br>(c)cyber security.
Norway	2018	2021		Art 2. The person who creates an intellectual work has copyright to the work, and is referred to as the originator. In this Act, intellectual works are understood as literary or artistic works of any kind, which are an expression of original and individual creative efforts, such as<br>l. computer programs<br><br>Art 41. Anyone who has the right to use a computer program can make copies of, change and process the program to the extent necessary to use the program in accordance with its purpose, including also to correct errors in the program. Anyone who has the right to use a database can take such actions as are necessary for access the database's content and normal use thereof.<br><br>Art 43. A published work can be performed in public and transmitted to the general public in teaching. If the author has handed over<br>a copy of a work of art or photographic work or if such works have been published, the work may be performed publicly and transferred<br>to the general public during teaching. In the case of vocational education, this section does not apply to either<br>a. performance and transmission of databases, or<br>b. other wired or wireless transmission to the public<br>Presentation and transfer in ordinary classroom teaching is considered to take place within the private area.<br><br>Art 71. Copyright to computer programs created by an employee during the performance of tasks covered by the employment<br>relationship, or according to the employer's instructions, passes to the employer. The same applies to access to changes to the

				work and transfer of the right. The provisions in the first and second sentence do not apply to rights under § 5 or where otherwise agreed.
Norway	2018			Art 1. Annex XI no. 5e of the EEA agreement (regulation (EU) 2016/679) on the protection of natural persons with regard to the processing of personal data and on the free movement of such data, and repealing Directive 95/46/EC (General Data Protection Regulation) will apply as a law with the adaptations that follow from Annex XI, Protocol 1 and the Agreement in general.<br><br>Art 2. This Act and the General Data Protection Regulation applies to processing of personal data wholly or partly by automated means and to processing other than by automated means of personal data which form part of a filing system or are intended to form part of a filing system. This Act and the General Data Protection Regulation do not apply when otherwise is stipulated in or pursuant to a statute.<br><br>Art 8. Personal data may be processed on the basis of Article 6(1) (e) of the General Data Protection Regulation if it is necessary for archiving purposes in the public interest, purposes related to scientific or historical research or statistical purposes. The processing shall be subject to the necessary safeguards in line with Article 89(1) of the General Data Protection Regulation.<br><br>Art 9. Personal data as mentioned in Article 9 (1) of the General Data Protection Regulation may be processed without consent from the data subject if the processing is necessary for archiving purposes in the public interest, purposes related to scientific or historical research or statistical purposes, and the public interest in having the processing take place, clearly outweighs the disadvantages for the individual. The processing shall be subject to the necessary safeguards in line with Article 89 (1) of the General Data Protection Regulation.<br><br>Before processing is performed based on the first subsection, the data controller shall consult the data protection officer pursuant to Article 37 of the General Data Protection Regulation or someone else who meets the requirements in Article 37 (5) and (6) and Article 38 (3) first and second sentence of the General Data Protection Regulation. This consultation shall assess whether the processing will meet the requirements in the General Data Protection Regulation and other provisions stipulated in or pursuant to this Act. The obligation to consult does not apply, however, if a data protection impact assessment has been conducted pursuant to Article 35 of the General Data Protection Regulation.

Turkiye	2017		Industrial Property Code No. 6769	Art 3(1). The following shall benefit from protection of this Code;<br>a) Citizens of Republic of Turkey,<br>b) Natural or legal entities domiciled or engaged in industrial or commercial activities within the borders of Republic of Turkey,<br>c) Persons who have the right of application according to the Paris Convention or Agreement Establishing the World Trade Organization.<br><br>Art 82(1). A patent shall be granted to the inventions in all fields of technology providing that the invention is new, involves an inventive step and is susceptible to industry.<br>Art 82(2). Below mentioned shall not be considered as inventions. In case the application for a patent or the patent itself are involved in the subjects or activities mentioned below, this subject only or the activity itself shall stay out of patentability:<br>a) Discoveries, scientific theories and mathematical methods;<br>b) mental acts, business activities or game related plans, rules and methods;<br>c) computer programs;<br>ç) products with aesthetical creations , belles-lettres, artworks and treatise;<br>d) presentation of the information.<br>Art 82(3). Below mentioned inventions shall not be granted a patent:<br>c) all treatment modalities including the diagnosis methods which are intended to be applied to human or animal bodies and surgical methods;
Turkiye	1951	1995	Law on Intellectual and Artistic Works No. 5846	Art 1. Within the meaning of this law, a work is any kind of intellectual and artistic product bearing the characteristics of its owner and which is considered a work of science and literature, music, fine arts or cinema under the following provisions.<br>Art 2. Works expressed in language and writing in any way and computer programs expressed in any form and their preliminary designs, provided that these lead to a program in the next stage;<br>Art 8. The owner of a work is the person who creates it.<br>Art 14. The owner of the work exclusively determines the presentation or non-presentation and the time and way of promulgation of a work. Only the owner of a work can give information on the content of a work, the whole or a substantial part of which has not become publicized or the main lines of which have not yet been introduced to the public. In case the presentation to the public or the way of publication of the work will humiliate the honour and prestige of its owner, the owner of the work, even if he had authorized someone else, may prohibit the introduction to the public or the publication of both the original and the adapted form of the work. The right to demand indemnity of the other party is reserved.

				Art 34. Creating selected and collected works from the published works of music, science and literature and publicized works of fine art, which are understandably aimed at training and education from their states and situations, by way of borrowing to the extent justified for the purpose is allowed.
Turkiye	1926	2003	Turkish Civil Code	Art 8. Every person is entitled to a vested right. Accordingly, all the persons are equal in using rights and fulfilling obligations within the legal limits.
